# The cGAS-STING pathway: a therapeutic target in diabetes and its complications

**DOI:** 10.1093/burnst/tkad050

**Published:** 2024-02-02

**Authors:** Wenjie He, Xingrui Mu, Xingqian Wu, Ye Liu, Junyu Deng, Yiqiu Liu, Felicity Han, Xuqiang Nie

**Affiliations:** Key Lab of the Basic Pharmacology of the Ministry of Education, Zunyi Medical University, No. 6 Xuefu West Road, Xinpu New District, Zunyi 563006, China; College of Pharmacy, Zunyi Medical University, No. 6 Xuefu West Road, Xinpu New District, Zunyi 563006, China; Key Lab of the Basic Pharmacology of the Ministry of Education, Zunyi Medical University, No. 6 Xuefu West Road, Xinpu New District, Zunyi 563006, China; College of Pharmacy, Zunyi Medical University, No. 6 Xuefu West Road, Xinpu New District, Zunyi 563006, China; Key Lab of the Basic Pharmacology of the Ministry of Education, Zunyi Medical University, No. 6 Xuefu West Road, Xinpu New District, Zunyi 563006, China; College of Pharmacy, Zunyi Medical University, No. 6 Xuefu West Road, Xinpu New District, Zunyi 563006, China; Key Lab of the Basic Pharmacology of the Ministry of Education, Zunyi Medical University, No. 6 Xuefu West Road, Xinpu New District, Zunyi 563006, China; College of Pharmacy, Zunyi Medical University, No. 6 Xuefu West Road, Xinpu New District, Zunyi 563006, China; Key Lab of the Basic Pharmacology of the Ministry of Education, Zunyi Medical University, No. 6 Xuefu West Road, Xinpu New District, Zunyi 563006, China; College of Pharmacy, Zunyi Medical University, No. 6 Xuefu West Road, Xinpu New District, Zunyi 563006, China; Key Lab of the Basic Pharmacology of the Ministry of Education, Zunyi Medical University, No. 6 Xuefu West Road, Xinpu New District, Zunyi 563006, China; College of Pharmacy, Zunyi Medical University, No. 6 Xuefu West Road, Xinpu New District, Zunyi 563006, China; Australian Institute for Bioengineering and Nanotechnology, The University of Queensland, Brisbane, QLD 4072, Australia; Key Lab of the Basic Pharmacology of the Ministry of Education, Zunyi Medical University, No. 6 Xuefu West Road, Xinpu New District, Zunyi 563006, China; College of Pharmacy, Zunyi Medical University, No. 6 Xuefu West Road, Xinpu New District, Zunyi 563006, China; Australian Institute for Bioengineering and Nanotechnology, The University of Queensland, Brisbane, QLD 4072, Australia; Joint International Research Laboratory of Ethnomedicine of Ministry of Education, Zunyi Medical University, No. 6 Xuefu West Road, Xinpu New District, Zunyi 563006, China

**Keywords:** STING, Pyroptosis, Reprogramming, Diabetic liver disease, Diabetic wound, Endoplasmic reticulum stress, inflammation, Cyclic GMP-AMP synthase

## Abstract

Diabetic wound healing (DWH) represents a major complication of diabetes where inflammation is a key impediment to proper healing. The cyclic GMP-AMP synthase (cGAS)-stimulator of interferon genes (STING) signaling pathway has emerged as a central mediator of inflammatory responses to cell stress and damage. However, the contribution of cGAS-STING activation to impaired healing in DWH remains understudied. In this review, we examine the evidence that cGAS-STING-driven inflammation is a critical factor underlying defective DWH. We summarize studies revealing upregulation of the cGAS-STING pathway in diabetic wounds and discuss how this exacerbates inflammation and senescence and disrupts cellular metabolism to block healing. Partial pharmaceutical inhibition of cGAS-STING has shown promise in damping inflammation and improving DWH in preclinical models. We highlight key knowledge gaps regarding cGAS-STING in DWH, including its relationships with endoplasmic reticulum stress and metal-ion signaling. Elucidating these mechanisms may unveil new therapeutic targets within the cGAS-STING pathway to improve healing outcomes in DWH. This review synthesizes current understanding of how cGAS-STING activation contributes to DWH pathology and proposes future research directions to exploit modulation of this pathway for therapeutic benefit.

Highlights

- Reviews role of the cGAS-STING pathway in diabetic complications and potential therapies.
- Summarizes crosstalk between cGAS-STING and nuclear factor kappa-B (NF-κB), Janus kinase-signal transducer and activator of transcription (JAK-STAT) and cellular senescence.
- Discusses regulators of cGAS-STING, such a ribosome collisions, DNA-dependent protein kinases, inhibitors, activators and metal ions.
- Relates cGAS-STING to diabetic wounds and predicts links to endoplasmic reticulum stress, pyroptosis and metabolic dysfunction.
- Proposes cGAS-STING mechanisms in wound fibroblasts and adipocytes.

## Background

Cells’ internal recognition and defense system against foreign genetic material contains an ancient and essential feature of the life system. The innate immune system coordinates the first line of defense of mammals [1]. Microbial infection is recognized by the innate immune system, which can trigger immediate defense and produce lasting adaptive immunity [2]. The innate immune system forms the first line of defense against pathogenic organisms. It detects pathogen-related molecular patterns [3,4].

These defenses depend on a large family of pattern recognition receptors (PRRs). PRRs trigger the intracellular signal cascade reaction, eventually leading to the expression of various pro-inflammatory molecules, coordinating the host’s early response to infection and being the prerequisite for the subsequent activation and formation of adaptive immunity [5]. In addition, the immune “dangerous model” that emerged in the 1990s attributed the activation of anti-tumor immune response to the non-physiological cell death and the subsequent release of specific molecules called damage-related molecular patterns (DAMPs) [6].

It has been found that various innate immune receptors can sense DAMPs [4,7]. The DAMPs produced by injured or dying cells promote aseptic inflammation, vital for tissue repair and regeneration, but can also lead to many inflammatory diseases [7]. Among them, DNA damage can be perceived by the innate immune system as DAMPs [8]. Initially, the immune system took the extracellular DNA as pathogen-related molecular patterns *in vivo* [9]. The sources of DNA fragments include mitochondrial (mt) DNA fragments produced by oxidative stress, cytoplasmic chromatin fragments (CCFs) produced by nuclear degradation during cell aging, DNA fragments produced when foreign bacteria or viruses invade the body, etc. These DNA fragments were subsequently detected by cGAS recognition receptors, resulting in a cGAS-STING immune response [1,10–13]. Studies have shown that the over-activated cGAS-STING signal axis is related to many inflammatory diseases. Partly blocking its conduction may be a potential therapeutic target for many diseases [14].

Type 2 diabetes (T2D), the most common type of diabetes, is characterized by insulin resistance due to overnutrition and innate immune activation [15,16]. The high glucose microenvironment in diabetes leads to immune response dysfunction, and the spread of invasive pathogens in diabetic patients cannot be effectively prevented. Therefore, people with diabetes are more susceptible to infection [17]. Originally, the immune system and metabolism complement each other and jointly maintain the conditions needed for the body’s survival [18,19]. Immune factors such as interleukin-1β (IL-1β) and IL-6 can help the physiological regulation of metabolism, and the immune system also needs the energy supply of metabolism [18].

However, long-term and exaggerated metabolic stress leads to harmful inflammatory reactions, induces inflammatory diseases and results in insulin resistance (IR) [19,20]. This chronic inflammatory state eventually leads to long-term complications of diabetes, including microvascular complications such as diabetic liver disease, diabetic nephropathy (DN) and neuropathy, and macrovascular complications such as cardiovascular and cerebrovascular diseases [19,21]. The cGAS-STING pathway plays an essential role in diabetic complications [22] and it has been reported increasingly in DN [23,24].

The cGAS-STING pathway has also been found in diabetic angiopathy, which is related to lipotoxicity-induced mitochondrial dysfunction [25]. Evidence shows that cGAS-STING is over-activated in diabetes and its complications [22,26–28], when the leakage of mtDNA in elderly mice is more serious [29]. This may be a protective mechanism because diabetic patients are more susceptible to infection [30], and interferon (IFN)-1α can also inhibit the development of diabetes [31]. Although inhibition of this pathway can alleviate the development of chronic inflammation in diabetes [23,26,32,33], there is no evidence that the degree of inhibition can ensure this protective mechanism will not be destroyed. Just like after knocking out STING, it reduces the IR induced by a high-fat diet (HFD) in peripheral tissues and overall glucose intolerance. However, STING deficiency also damages the glucose-stimulated insulin secretion of β cells [34].

Additionally, the absence of STING partially attenuates type I IFN gene markers in the pancreatic islets, but does not suppress insulitis, instead promoting T cell infiltration in peripheral lymph of Non-obese diabetic (NOD) mice. Thus, the STING pathway may unexpectedly inhibit the number of T cells in diabetes [35]. Oxidative stress and mitochondrial dysfunction are contributing factors to the pathogenesis of aging [36]. The hyperglycemic microenvironment of diabetes causes cells to suffer from oxidative stress [37–39], and cGAS-STING is also involved, especially when the leakage of mtDNA from aging cells is more pronounced [29].

Research shows that inhibiting signal transduction of cGAS-STING can inhibit cell senescence from achieving the effects of maintaining the integrity of the nuclear envelope and reducing the release of senescence-associated secretory phenotype (SASP) [40,41]. While inhibiting this pathway may potentially produce anti-aging effects by reducing SASP production, its function cannot be generalized given the susceptibility of diabetic patients, and its mechanism of action under different pathological conditions requires further elucidation. In this review, we will briefly introduce the mechanism of the cGAS-STING pathway, including some cellular homeostasis and regulatory factors, and the research progress of cGAS-STING in diabetic complications and diabetic wound healing. Because there are few studies on the cGAS-STING pathway and diabetes, with some indirect literature reports, we can still observe that the cGAS-STING pathway is inextricably linked with diabetes and its complications. This review may provide valuable insights into the precise treatment of diabetes and may help guide future research.

## Review

### Molecular mechanism of the cGAS-STING pathway

**Figure 1:**
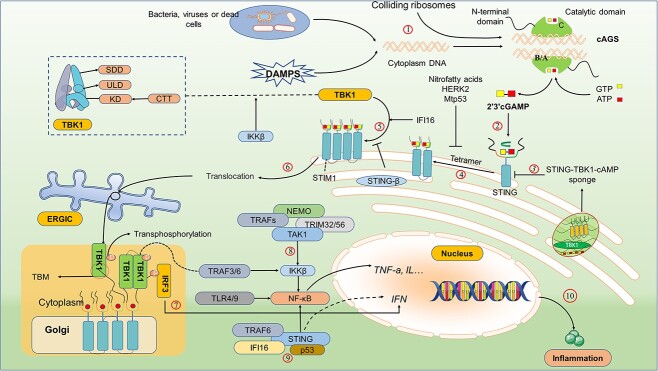
Molecular mechanism of the cGAS-STING pathway. Bacteria and viruses, or some DAMPs, produce abnormal DNA that activates cGAS, producing cGAMP. cGAMP binds to STING transmembrane protein on the endoplasmic reticulum, a process inhibited by spongy cGAMP-STING-TBK1. After being activated by cGAMP, STING forms dimers and recruits TBK1 for ubiquitination. However, this ubiquitination is blocked by nitro-fatty acids, hERK2 and Mtp53. After activation, the connection of STING to STIM1 is cut off, and STING is transferred to the ERGIC together with TBK1, which in turn undergoes trans-autophosphorylation activation of TBK1, followed by tetramerization of STING to provide a phosphorylation site for IRF3 and subsequent phosphorylation of IRF3 by TBK1, producing IFN. Additionally, the NF-κB pathway is also activated by TBK1. Some inflammatory factors produced by these processes can lead to the production of senescence phenotype and the infiltration of inflammatory cells. *cGAS* cyclic GMP-AMP synthase, *B/A* DNA binding site B/A, *C* DNA binding site C, *cGAMP* 2′3′cyclic GMP–AMP, *STING* stimulator of interferon genes, *TBK1* TANK-binding kinase 1, *ULD* ubiquitin-like domain, *SDD* scaffold/dimerization domain, *KD* kinase domain, *IRF3* interferon regulatory factor 3, *DAMPS* damage-related molecular patterns, *hERK2* human extracellular signal-regulated kinase 2, *Mtp53* mutated p53, *STIM1* stromal interaction molecule 1, *ERGIC* ER-Golgi intermediate compartment, *TRAF* TNF receptor associated factor, *TLR* toll-like receptor, *NEMO* NF-κB essential modulator, *IFI16* interferon gamma induction 16, *IL* interleukin, *IFN* interferon

cGAS is a PRR containing a nucleotide transferase and two major DNA binding domains. Free DNA (usually >40 bp in cells) binds to a free cGAS, forming a cGAS dimer and providing a binding site for GTP and ATP [1,42–44]. Gene mutation caused by base-sequence change will not increase the binding with cGAS. However, the cytoplasmic DNA caused by oxidation is difficult to hydrolyse, which provides more opportunities for cGAS to bind with cytoplasmic DNA fragments (Figure 1) [45].

cGAS consists of a two-lobed catalytic domain and an extended amino-terminal (N-terminal) domain. It recognizes and is activated by DNA ligands (canonically single-stranded or double-stranded molecules longer than 40 bp) and assembles into a dimer, in which both cGAS protomers are bound to their own DNA ligand, with the DNA strands sandwiched between the two cGAS protomers. Each protomer has two principal DNA binding sites, A and B. In the dimer, the DNA molecules are bound to site A of one cGAS protomer and to site B of the respective other protomer. Interestingly, a third DNA binding site, termed site C, was recentlydentified and shown to promote mesh-like interactionsor even formation of lattice-like structures in such condensates. In normal human mitosis, the N-terminal of cGAS is over-phosphorylated to prevent the chromatin of exposed DNA from being recognized, and deoxyribonucleases of double-stranded DNA (dsDNA), such as extracellular enzyme I, DNase II and DNase 3 (also called TREX1) in the cytosol are digested to ensure that cytoplasmic DNA will not accumulate and trigger cGAS [46–49]. Trex1-specific knockout decreased the activity of rapamycin complex 1, which may be mediated by TANK (TRAF family member associated NFkappaB activator)-binding kinase 1 (TBK1), resulting in metabolic imbalance and systemic inflammation involving the cGAS-STING pathway (Figure 1) [50]. In addition, the inhibition of the cGAS-STING pathway is also limited by the natural vesicles of the Golgi apparatus (Figure 1) [51].

Ribosome collision leads to the stagnation of ribosomes, resulting in translation abnormalities and the generation of many harmful truncated peptides to cells. At this time, cells will initiate conservative methods of ribosome related protein quality control (RQC) to cause the decay of ribonucleic acids. [52–54]. Interestingly, RQC includes ASC-1 complex (ASCC) protein. During translation stress, the early disruption of RQC leads to the continuous collision of ribosomes. In contrast, the complete ribosome can directly act on cGAS and stimulate cGAS together with DNA to activate the subsequent STING-interferon regulatory factor 3 (IRF3)-interferon-stimulated gene. It is worth noting that cGAS has higher priority in binding with colliding ribosomes than DNA, but this process still needs the participation of cytoplasmic DNA [55].

When DAMPs or bacterial/viral factors occur, cGAS is activated by abnormal cytoplasmic DNA and then combines with ATP and GTP to form 2'3'-cyclic GMP-AMP (cGAMP) [1,56]. cGAMP is a potent affinity ligand for STING, which modifies and triggers the conformational change of STING’s ligand binding domain (LBD), thus enhancing its oligomerization activation. The activation of STING provides a model basis for the self-transphosphorylation of TBK1 (Figure 1) [57,58].

When the butterfly-shaped LBD in STING merged with cGAMP, the LBD rotated 180 degrees clockwise relative to the transmembrane domain, observed from the top to the membrane [59]. Subsequently, the binding between STING and stromal interaction molecule 1 (STIM1), where the endoplasmic reticulum (ER) resides, is destroyed, which leads to STING translocation, C-terminal tail (CTT) release at the carboxyl-terminal and tetramer formation. The released CTT can bind and promote the activation of TBK1 [11,60].

After STING activation, the tail of its C-terminal convenes and phosphorylates TBK1. TBK1 is then activated by its dimerization. Activated TBK1 causes STING to form a tetramer that provides a binding site for IRF3. TBK1 then phosphorylates and activates IRF3 [11,60,61]. TBK1 is also proved to be an inhibitor of kappa B kinase (IKK) dependent on STING activation. However, it must be mediated by TNF receptor associated factor (TRAF)3/6 [62]. The absence of TBK1 causes a significant decrease in nuclear factor kappa-B (NF-κB) expression in macrophages [63]. Interferon-inducible 16 (IFI16) can also moderate the immune response by activating the STING-NK-κB pathway unconventionally [64]. Downstream signals from STING also trigger the Janus kinase–signal transducer and activator of transcription (JAK-STAT) pathway, induce ER stress (ERS) and autophagy, and spread to the surrounding cells (Figure 1) [42]. This path also has a feedback program, whereby the mutation of STING may result in the release of its C-terminal tail, thereby inhibiting the polymerization of STING [65].

The adenosine and guanosine components of cGAMP are linked by two phosphodiester links, and there are multiple isomers, including 2'3'-cGAMP and 3'3'-cGAMP. Intracellular cGAMP concentration determined translocation or condensation of STING, but no 2'3'-cGAMP hydrolase was identified in the cells. A spherical STING membrane bio-dense (phase separator or condensate) in the ER acts as a phase separator or “STING-TBK1-cGAMP sponge”, and Mn^2+^ can enhance this effect, limit the combination of STING and TBK1 and avoid the over-activation of innate immunity (Figure 1) [66]. Furthermore, STING-β, a newly discovered transcript isoform, is also a negative feedback regulatory mechanism of cGAS-STING; it can isolate the binding of STING-α to cGAMP and TBK1, etc., thereby negatively regulating the production of IFN [67].

IFI16 can also mediate the immune response by activating the STING-NK-κB pathway in a non-classical manner [64]. Ubiquitination is the foremost step for the activation of NF-κB. Etoposide-induced DNA damage in human keratinocytes leads to the binding of IFI16 to Ser15 of p53 and STING through ataxia telangiectasia mutated transmission, while TRAF6 also leads to the ubiquitination of K63 in STING through the transient binding of IFI16. Together, they assembled and promoted the atypical activation of STING, leading to the activation of NF-κB [68].

The activation of NF-κB essential modulator (NEMO) mediated by dsDNA requires binding to the E3 ubiquitin ligase of tripartite motif (TRIM)32/56. Then NEMO with the K63 ubiquitin chain was able to activate TRAFs, and TAK1 is then activated by TRAFs. Finally, the activated TAK1 is phosphorylated to activate IKKβ, which was proved to be necessary for the activation of NF-κB and TBK1, and finally affected the production of TNF-α and IFN [69]. Remarkably, evidence shows that TBK1 may not be necessary for the activation of NF-κB induced by STING [63]. Downstream signals from STING also trigger the JAK–STAT pathway, induce ERS and autophagy, and eventually spread to the surrounding cells (Figure 1) [42]. In short, the activation of cGAS-STING leads to an inflammatory crosstalk reaction.

### Inflammatory pathway related to cGAS-STING

#### The cGAS-STING pathway and the NF-κB pathway

Human and murine STING activation also trigger NF-κB pathway activation, albeit to a lower extent compared with other PRR cascades [70]. Ataxia telangiectasia mutated (ATM) and poly (ADP-ribose) polymerase 1 (PARP-1) were activated after DNA damage induced by etoposide, which made p53, ATM and TRAF6 gather on STING and catalyzed the formation of the K63-linked ubiquitin chain on STING, resulting in p65 phosphorylation of NF-κB [71]. TBK1 belongs to the family of non-standard inhibitory nuclear factor κB (IκB) kinases (IKK) that mediates the inflammatory pathway of NF-κB by activating IKK [62,72,73].

In adipocytes, AMP-activated protein kinase (AMPK) is a mechanism of energy consumption, and its activation can increase the expression of TBK1 through UNC-52-like kinase 1 (ULK1). However, the activation of TBK1, in turn, can also inhibit AMPK, inhibit respiration and increase energy storage. In obesity, the activation of TBK1 will inhibit NF-κB-inducing kinase (NIK), thus inhibiting NF-κB from achieving the effect of reducing inflammation, showing that TBK1 plays a bidirectional role in energy storage and metabolism to achieve a balance between energy metabolism and inflammation [74–76].

**Figure 2:**
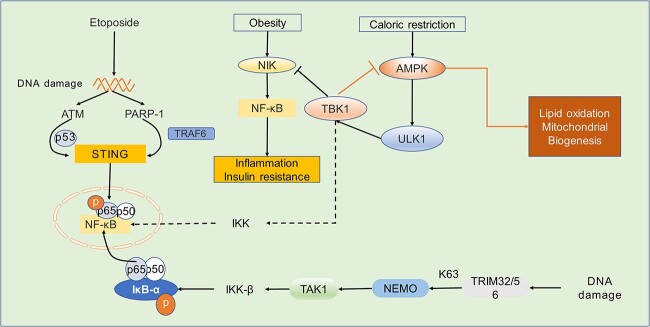
cGAS-STING pathway and the NF-κB pathway. DNA damage can be recognized by ATM, PARP-1 and c32/56, leading to activation of ubiquitination of STING or NEMO, resulting in activation of the NF-κB signaling pathway. TBK1 may also induce activation of IKK, which activates NF-κB. in addition, TBK1 plays a dual role in metabolism. During energy consumption, the AMPK signaling pathway can activate TBK1 via ULK1, which in turn acts as a feedback system to inhibit AMPK, thereby suppressing caloric expenditure. Over-activated TBK1 in turn suppresses inflammation and insulin resistance by inhibiting obesity-mediated activation of NIK and NF-κB. *ATM* ataxia telangiectasia mutated, *PARP-1* poly (ADP-ribose) polymerase1, *STING* stimulator of interferon genes, *TRAF* TNF receptor associated factor, *IKK* inhibitor of kappa B kinase, *IκB* inhibitory nuclear factor- κB, *TAK1* transforming growth factor-beta-activated kinase 1, *NIK* NF-κB-inducing kinase, *AMPK* AMP-activated protein kinase, *ULK1* UNC-52-like kinase 1, *NEMO* NF-κB essential modulator, *TRIM* tripartite motif, *K63* lysine 63, *cGAS* cyclic GMP-AMP synthase

Inhibiting IKK by inhibiting TBK1 and increasing AMPK induction can reduce inflammation and increase energy metabolism, which may improve obesity [77]. The existence of TBK1 is fundamental. A lack of TBK1 in humans leads to TNF-induced cell death-driven autoinflammation, while a lack in mice is fatal [78]. Previous studies have found that TBK1 works upstream of NF-κB [62,79], but there is evidence that CTT is not needed for this procedure, which means that STING’s recruitment and activation of TBK1 may not affect the degree of activation of NF-κB [70,80]. Recent studies have also shown that there is still a normal STING-NF-κB response in macrophages lacking TBK1 [63]. Therefore, activation of NF-κB downstream of cGAS-STING may not be due to catalysis by TBK1, so it is speculated that STING-dependent NF-κB activation might be controlled by signals within the LBD from oligomerized STING (Figure 2) [1]. The linkage among STING, TBK1 and NF-κB is still vague, so further research is needed to uncover their relationship in the future.

#### The cGAS-STING pathway and the JAK–STAT pathway

The JAK–STAT signal pathway is related to the pathogenesis of various inflammatory or immune diseases. Combining cytokines and receptors leads to JAK’s activation and phosphorylation and receptors’ phosphorylation. When IFN-α/β binds to its receptor, the related JAK1 phosphorylation is activated, and the phosphorylation activates the corresponding STAT1/2/3/4, which then promotes the gene’s transcription and produces a cascade reaction [81,82].

**Figure 3:**
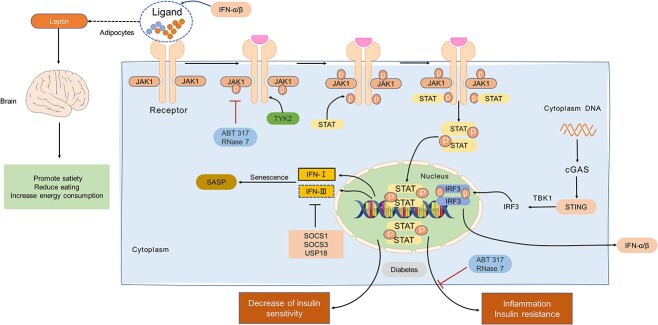
cGAS-STING and JAK–STAT pathways. JAK is activated after receiving the ligand. When the ligand leptin produced by adipocytes is transmitted to the brain, the brain will transmit the signal to promote energy metabolism and reduce appetite. When the ligand is IFN produced by the cGAS-STING pathway, cascade reactions will generate IFN-I/III and finally generate SASP; this pathway is inhibited by cytokine SOCS1/3 and USP18. Furthermore, in diabetes, JAK–STAT activation will lead to decreased insulin sensitivity and obesity-related inflammation, but ABT317 and RNase7 can inhibit it. *cGAS* cyclic GMP-AMP synthase, *cGAMP* 2′3′cyclic GMP–AMP, *STING* stimulator of interferon genes, *TBK1* TANK-binding kinase 1, *IRF3* interferon regulatory factor 3, *JAK* Janus kinase, *ABT* 317 a selective inhibitor of JAK1, *TYK2* tyrosine kinase 2, *STAT* signal transducer and activator of transcription, *IFN* interferon, *SOCS* cytokine signal transduction inhibitor, *USP18* ubiquitin-specific peptidase 18, *SASP* senescence-associated secretory phenotype, *cGAS-STING* cyclic GMP-AMP synthase-stimulator of interferon genes

However, the signaling is negatively regulated by the suppressor of cytokine signaling1 (SOCS1), SOCS3 and ubiquitin-specific peptidase 18 (USP18), which is very important for regulating insulin sensitivity [82–85]. Interferon has type I IFN and type III IFN, but physiological concentrations of SOCS3 and USP18 do not regulate the production of type III IFN, and its STAT phosphorylation process allows for durable interferon-stimulated gene transcription (Figure 3) [85].

JAK–STAT plays a vital role in obesity or diabetes. For adipocytes, STAT3/5 in leptin receptors can feed back to the brain to promote satiety, reduce eating and increase energy consumption. At the same time, STAT3 in liver cells may also partially inhibit lipogenesis mediated by sterol regulatory element binding protein, thus reducing fatty liver degeneration [86]. However, under chronic inflammation of diabetes, JAK–STAT in immune cells is continuously activated, leading to decreased tissue insulin sensitivity. So far, the effects of JAK–STAT in macrophages, natural killer (NK) cells and T cells on obesity-related inflammation and glucose homeostasis have been studied, and inhibition of the JAK–STAT pathway can partially improve diabetes inflammation and insulin resistance [87–89].

Recent studies have demonstrated that the use of the JAK1 selective inhibitor ABT-317 to treat type 1 diabetes reduces the production of proinflammatory factors in T cells within islets, resulting in effective control of blood sugar and a safer treatment option compared to schemes lacking IFN-γ receptor alone [90]. Another study indicated that RNase 7 might also prevent infection and inflammation of diabetic cystitis by inhibiting the JAK–STAT signaling pathway [91].

Cells in diabetes and the elderly undergo aging processes, and when infected with COVID-19, the JAK–STAT pathway in aging cells can be overactivated by the synergistic effect of TNF-α and IFN-γ, leading to further aggravation of SASP expression and inflammation [92]. In T1DM, differential expression of the cyclic RNA-I_circ_0060450 was detected, which was found to act as a sponge for miR-199a-5p to release protein tyrosine phosphatase 2 from its target gene Src homology 2 through encoding the tyrosine-protein phosphatase non-receptor type 11 geneI [93]. Molecular pathways such as cGAS-STING can induce the production of IFN, which combines with tyrosine kinase 2 and JAK1 to activate two kinases through trans-phosphorylation, targeting STAT to produce a series of tyrosine phosphorylation events, and ultimately inducing the production of IFN autocrine and paracrine signals (Figure 3) [85].

JAK–STAT is essential in promoting the inflammatory development of diabetes and obesity. Therefore, when cGAS-STING is activated, the expression of IFN-α/β increases and the JAK–STAT pathway downstream of the IFN-α/β receptor also increases. Conversely, inhibition of the cGAS-STING pathway may have the effect of suppressing JAK–STAT, thus achieving the goal of alleviating the development of chronic inflammation of diabetes.

#### The IFI16-STING and DNA-dependent protein kinase-STING pathway

IFI16, a PYHIN protein, is another innate immune sensor of intracellular DNA. It can irregularly recruit and activate STING and induce the expression of IFN, which is very important for immune regulation mediated by cGAS-STING [94,95]. IFI16 can also sense viral RNA through retinoic acid-induced gene I receptor [96]. According to research, IFI16 can not only amplify the function of cGAS but also promote cGAMP production under DNA damage, and the dimerization and phosphorylation of STING in macrophages are also controlled by IFI16, which leads to the activation of downstream STING-TBK1 [97].

Similarly, IFI204 seems to cooperate with cGAS to sense dsDNA and activate the STING-dependent type I IFN pathway, but few studies exist [98]. It is worth noting that IFI16 does not affect the production of cGAMP in keratinocytes but directly binds to STING [99,100]. However, not all human cells can produce IFN immune resistance in response to external DNA, even if the DNA sensors cGAS and IFI16 and cGAS-STING downstream pathways are intact, e.g. human B cells [101]. IFI16 binds to STING by its pyrin domain [97,102]. After activating STING, it not only activates the TBK1-IRF3 pathway but also induces the activation of NF-κB through TRAF6 [71]. In addition, when IFN is overexpressed, overactivated STING triggers ubiquitinated protease degradation of IFI16, thus forming a negative feedback regulation mechanism [103].

**Figure 4:**
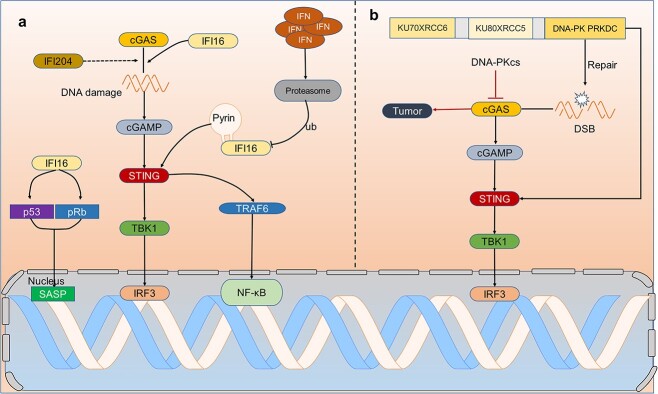
Other media for activating STING. (**a**) IFI16 and IFI204 are also involved in the cGAS-STING signaling pathway, which enhances the ability of cGAS to bind DNA and is required for STING activation as a means to enhance IRF3 and NF-κB activation. In turn, there is also negative feedback regulation between IFI16 and cGAS-STING, i.e., excess product IFN promotes ubiquitinated degradation of IFI16 by the proteasome. Finally, IFI16 also promotes the activation of p53 and p-RB, which leads to the production of SASP, resulting in cellular senescence. (**b**) As a mediator of DSB repair, DNA-PK activates STING but inhibits cGAS, resulting in DNA-PK-STING-IRF3 activation. *cGAS* cyclic GMP-AMP synthase, *cGAMP* 2′3′cyclic GMP–AMP, *STING* stimulator of interferon genes, *TBK1* TANK-binding kinase 1, *IRF3* interferon regulatory factor 3, *IFI16* interferon-inducible 16, *IFI204* interferon gamma induction 204, *Ub* ubiquitination, *p53* tumor protein P53, *pRB* phosphorylated retinoblastoma, *SASP* senescence-associated secretory phenotype, *TRAF* TNF receptor-associated factor, *DSB* DNA double-strand breaks, *DNA-PKcs* DNA-dependent protein kinase

Alternatively, or additionally, IFI-16 can promote and enhance the cell growth inhibition mediated by p53 and phosphorylated retinoblastoma (pRB), to further enhance the expression of SASP [98,102]. In short, an in-depth study on the physiological function of IFI-16 in metabolic diseases will enable us to understand better the production of IFN mediated by the cGAS-STING pathway and may provide new therapeutic targets for immune diseases (Figure 4a).

Like IFI16, the DNA-dependent protein kinase (DNA-PK) complex appears to be involved in cGAS-STING [100]. The DNA-PK complex is composed of KU70XRCC6, KU80XRCC5 and DNA-PK PRKDC, the catalytic subunit of DNA-PK, which participate in the repair of DNA double-strand breaks (DSB) mediated by nonhomologous end-joining, and which is an alternative way to stimulate the production of type I IFN and plays a role in controlling nucleic acid-dependent inflammation [104,105]. Studies have shown that DNA-PK acts on STING-mediated IFN production. However, the DNA-PK complex may inhibit the activity of the cGAS enzyme, which is very important for DNA repair to maintain the stability of genes, because cGAS may inhibit DNA repair and thus promote the occurrence of tumors [100,106,107]. These observations indicate that DNA-PK-STING-IFN might be another immune pathway different from cGAS-STING, although other studies have shown that DNA-PK may also operate independently of STING (Figure 4b) [108].

In conclusion, the cGAS-STING pathway can be regulated by various mediators. STING-mediated inflammation results from crosstalk among multiple pathways rather than just the induction of DNA by cGAS. In the future, more experiments are needed to explore and clarify the cross-cutting relationship between cGAS-STING and other pathways.

### cGAS-STING and ERS

**Figure 5:**
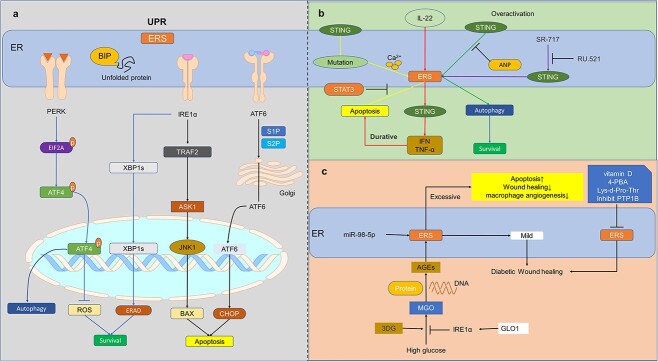
Relationship between cGAS-STING and ERS in diabetic wounds. (**a**) When unfolded proteins increase within the ER, it causes ERS, and the main initiating form of ERS is UPR, and the UPR reaction mainly consists of three reactions, i.e., PERK-EIF2A, IRE1α, and ATF6. When an increase in unfolded proteins is detected, the BIP segregates and leads to activation of the three transmembrane proteins of PERK, IRE1α, and ATF6, which ultimately leads to apoptosis and autophagy reaction occurrence. (**b**) Abnormal over-activation of IL-22 or STING induces ERS, which in turn performs UPR, followed by a cascade of events such as ERAD, translational repression, and other events to alleviate ERS. However, when ERS is prolonged, it leads to apoptosis, but mild UPR promotes proper protein folding and facilitates cellular survival. After ERS, ER-phagy occurs, which restores the ER to its original state and recycles catabolic proteins. Autophagy is a cellular emergency program that inhibits apoptosis caused by overactivated ERS. and STAT3 also has this role. Interestingly, STING overactivation also activates ERS and autophagy, but prolonged activation leads to apoptosis. However, ANP inhibited apoptosis or ERS caused by cGAS-STING overactivation. (**c**) In the presence of 3DG, MGO is readily generated in high glucose microenvironments and subsequently generates AGEs along with DNA short chains and proteins. AGEs activate ERS as does miR-98-5p, and mild ERS facilitates wound healing, however, sustained ERS in diabetic wounds leads to delayed healing. GLO1 inhibits the generation of AGEs through IRE1α, thereby inhibiting ERS. In addition, inhibition of vitamin D, 4-PBA, lys-D-pro-thr, and PTP1B inhibits ERS, thereby promoting diabetic wound healing. *cGAS* Cyclic GMP-AMP synthase, *cGAMP* 2′3′cyclic GMP–AMP, *STING* stimulator of interferon genes, *TBK1* TANK-binding kinase 1, *IRF3* interferon regulatory factor 3, *IFI16* interferon-inducible 16, *IFI204* interferon gamma induction 204, *ER* endoplasmic reticulum, *ERS* endoplasmic reticulum stress, *UPR* unfolded protein response, *PERK* protein kinase RNA-like ER kinase, *IRE1α* inositol-requiring enzyme 1 α, *ATF4/6* activating transcription factor 4/6, *TRAF* TNF receptor associated factor, *ASK1* apoptosis signal regulating kinase 1, *JNK1* c-Jun N-terminal kinase 1, *CHOP* C/EBP homologous protein, *ERAD* endoplasmic reticulum (ER)-associated degradation, *EIF2A* eukaryotic initiation factor 2A, *S1P* site 1protease, *S2P* site 2 protease, *ANP* atrial natriuretic polypeptide, *SR717* STING agonist, *RU 521* selective cGAS inhibitor, *STAT3* signal transducer and activator of transcription 3, *TNF-α* tumor necrosis factor alpha, *4-PBA* 4-phenyl butyric acid, *AGEs* advanced glycation end products, *MGO* methylglyoxal, *3DG* 3-deoxyglucose ketone, *GLO1* glyoxalase 1

Under ERS, unfolded protein increased and membrane expansion occurred, activating the unfolded-protein response (UPR). The UPR assists in unfolding folded protein correctly or other related protein degradation processes via three ER transmembrane protein-mediated pathways. The pathways consist of protein kinase RNA-like ER kinase (PERK), activating transcription factor 6 (ATF6) and inositol-requiring enzyme 1 (IRE1). PERK is generally triggered by phosphorylation of eukaryotic translation initiation factor 2α (EIF2α), hampering protein translation. IRE1α is an endonuclease associated with ER-related degradation (Figure 5a) [109].

Three transmembrane protein receptors can also be triggered by self-dimerization or self-phosphorylation, inhibited by the ER chaperone protein binding protein (BIP) binding glucose regulatory protein GRP78 or HSPA5 (Figure 5a) [110]. BIP detaches from transmembrane protein emergency receptors and assists in protein folding [111]. BIP separates from ATF6, and the free ATF6 assists the transport vesicle of coatomer protein complex II (COPII) to the Golgi apparatus [110], which may be an enormous influence on STING. Afterward, some reticular bodies undergo lysosomal degradation via autophagy or macroautophagy to recover and restore the morphology and function of ER, known as ER-phagy [109,112]. If ERS cannot be restored, cells will generate C/EBP (CCAAT/enhancer-binding protein)-homologous protein (CHOP, also known as GADD153) apoptosis protein, allowing the transcription of Bcl-2-interacting mediator of cell death, weakening the expression of Bcl-2 and creating a JNK signal, jointly inhibiting apoptosis (Figure 4a) [110].

ERS plays an essential role in diabetes. Methylglyoxal (MGO) is a highly active dicarbonyl species formed during hyperglycemia in a high-glucose microenvironment. It can create advanced glycation end-products (AGEs) with protein, DNA, etc. [113–115]. Studies have found the AGEs can trigger ERS of skin fibroblasts that finally leads to apoptosis due to the activation of the PERK-eIF2α pathway and caspase-12 [113]. The presence of MGO delayed wound healing in diabetic mice. At the same time, its scavenger, glyoxalase 1, could promote wound healing by enhancing the activity of IRE1α [116]. UPR is a way of cell self-salvation in ERS. Studies have shown that mild UPR can promote wound healing, while accumulated ERS hinders wound healing [117]. The prolonged presence of ERS in diabetic wound healing (DWH) caused a phenotypic reduction in macrophage angiogenesis and slowed wound healing (Figure 5) [118]. NADPH oxidase 4, a homolog of reactive oxygen species (ROS), can also trigger ERS of skin fibroblasts through oxidative stress.

ERS can induce the growth arrest of apoptosis markers and the production of DNA damage-inducing gene 153 (GADD153). Interestingly, combining AGEs modified by AGE 3-deoxyglucose ketone with CHOP can activate caspase-3 and induce apoptosis [119]. Besides, it was found in diabetic mice that the expression of miR-98-5p in keratinocytes also increased the level of ERS, thus reducing cell proliferation and inducing apoptosis [120]. Similarly, in diabetic keratinocytes, the high-sugar microenvironment induces oxidative stress and ERS. Studies have found that Lys-d-Pro-Thr, a tripeptide derived from α-melanocyte-stimulating-hormone, can inhibit this stress and may be used for future DWH [121].

Alternatively (or in addition), protein tyrosine phosphatase 1b (PTP1B) appeared in the ER membrane and was over-expressed in DWH. Studies have shown that inhibition of PTP1B assists in controlling ERS and improving wound healing [122]. Surprisingly, vitamin D and 4-phenyl butyric acid can also reduce the ERS of diabetic mice caused by AGEs, thus promoting wound healing (Figure 5) [117,123]. These shreds of evidence show that ERS is critical to the occurrence of DWH, and if ERS reaction can be inhibited, it is possible to alleviate the development of DWH. ERS is implicated in other diabetes-related complications which will not be expounded upon in this study.

ERS has been suggested to be associated with the cGAS-STING signaling pathway (Figure 5c). STING is a unique protein receptor residing on ER, and the positional relationship has already indicated the correlation between STING and ERS. There is increasing evidence for a close relationship between SITNG and ERS. For example, STING mutations can lead to excessive ERS and cell death, as Wottawa *et al*. summarized, which may be due to excessive signaling of STING disrupting calcium homeostasis [124,125]. The latest research has also found that in inflammatory bowel disease, it is confirmed that ERS and autophagy pathways control the activation of STING-dependent signaling, which is regulated by IL-22 [126]. The atrial natriuretic polypeptide has also been found to inhibit the STING signal in epithelial cells and reduce ERS and ERS-induced autophagy caused by the over-activation of STING in ulcerative colitis [127]. In addition, STING has been defined as an important signal that ERS is closely associated with in cardiac hypertrophy. [128] The same is true of hepatocytes and macrophages, but activation of autophagy can prevent the cell death caused by this process [129,130]. Macrophages in obese mice have also been used to show that ER-associated degradation can also regulate the size of the STING pool by ubiquitinating and targeting the proteasome for STING degradation, thereby mediating the STING immune response [131]. In addition to the rescue pathway of autophagy, STAT3 in hepatocellular carcinoma cells can inhibit ERS-mediated STING activation and apoptosis [132]. After activation of cGAS-STING by SR-717 in alveolar epithelial cells, ERS was up-regulated, while RU.521 specifically inhibited cGAS signaling and reduced ERS [133]. This may be the result of a disruption of STING-mediated calcium homeostasis in T cells and chronic activation of ERS, where immune function is disrupted resulting in some lung diseases [125]. Such evidence indicates that ERS and STING may be mutually regulated in different pathological conditions.

Nevertheless, the exact interplay between these two processes is not yet fully elucidated in the complications of diabetes, necessitating further investigation (Figure 5c).

### cGAS-STING and pyroptosis

**Figure 6:**
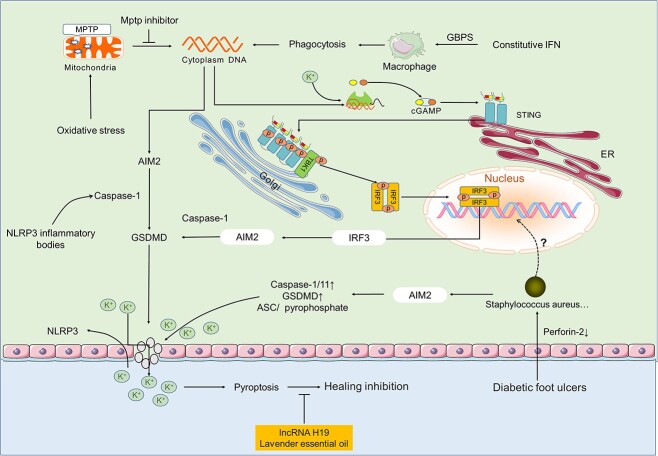
cGAS-STING and pyroptosis. Oxidative stress leads to the production of mPTP and increases membrane permeability, resulting in the generation of mtDNA. Additionally, bacteria ingested by macrophages can undergo cleavage by guanylate binding proteins, leading to the production of cDNA. These events activate the cGAS-STING pathway, triggering the production of IFN. Abnormal cDNA and IFN are detected in AIM2 inflammasomes, which activate caspase-1 cleavage and AIM2 activation, resulting in the production of GSDMD perforation protein and the subsequent release of potassium ions (K^+^), ultimately inducing pyroptosis. The released K^+^ can further activate NLRP3 inflammasomes. Notably, K^+^ can also inhibit the activity of cGAS-STING during focal cell death by promoting the binding of cGAS to DNA. *cGAMP* 2′3′Cyclic GMP–AMP, *STING* stimulator of interferon genes, *MPTP* mitochondrial permeability transition pore, *GBPS* guanylate-binding proteins, *AIM2* absent in melanoma 2, *GSDMD* gasdermin D, *IRF3* interferon regulatory factor 3, *NLRP3* NOD-like receptor thermal protein domain associated protein 3, *ASC a*poptotic speck protein, *ER* endoplasmic reticulum,* cGAS-STING* cyclic GMP-AMP synthase-stimulator of interferon genes

There are several methods of cell death, such as apoptosis, necrosis, autophagy induction, etc. [134,135]. Pyroptosis is a recently proposed new cell death method [135]. Pyroptosis is mainly caused by the inflammatory body absent in melanoma 2 (AIM2), which was initially a tumor inhibitor [136]. cGAS-STING is closely associated with pyroptosis mediated by AIM2. Lipopolysaccharide (LPS) may also increase the expression of IFN through the cGAS-STING pathway Pyroptosis occurs, K^+^ is essential for enhancing cGAS binding to dsDNA, and Pyroptosis occurs with a decrease in intracellular K^+^, whereas LPS no longer induces the production of IFN, which demonstrates the specificity of K^+^ for cGAS. K^+^ is essential for strengthening the binding of cGAS to dsDNA. Still, its mechanism of promoting binding requires further investigation (Figure 6). Studies have shown that cytoplasmic DNA triggers the NOD-like receptor thermal protein domain associated protein 3 (NLRP3) inflammatory body and cGAS-STING, which share upstream components [137,138].

Pyroptosis and K^+^ leakage occurred after STING was activated. The presence of K^+^ triggered NLRP3 [139], forming the signaling pathway of STING-NLRP3 [137]. Notably, oxidative stress aids in opening the mitochondrial permeability transition pore (mPTP) and activating the STING-NLRP3 axis, leading to pyroptosis. Then, the generated dsDNA will not only trigger the cGAS-STING axis, produce excessive IFN and cause harmful inflammation but also activate AIM2 inflammatory body, induce caspase-1 to cut its C-terminal inhibitory structure, and then produce IL-1β, IL-18 and the pore-forming protein gasdermin D (GSDMD). GSDMD was observed to create a hole in the cell membrane to release K^+^ and IL, but the lack of K^+^ hindered the fusion of cGAS and dsDNA, reduced the IFN produced by the cGAS-STING signal and created a feedback path [140,141].

Inhibition of mPTP to reduce the cytoplasmic leakage of mtDNA can effectively slow down the pyroptosis mediated by NLRP3 inflammation and the development of an inflammatory environment [142]. However, not all cells that trigger GSDMD face death [143]. When pyroptosis occurs, cell death depends on the pre-existing IFN state in cells (Figure 6) [144].

In podocytes under high glucose or diabetic pathological conditions, the markers caspase-11 and GSDMD of pyroptosis increased (Figure 6) [145]. This indicates that pyroptosis appears in DN. Diabetic foot ulcers (DFU) patients are vulnerable to recurrent *Staphylococcus aureus* infection and persistent chronic inflammation. Perforin-2 plays an important role in preventing skin infection and the spread of *S. aureus* in mice. When *S. aureus* infects diabetes patients, perforin-2 is significantly inhibited and triggers the activation of AIM2 inflammatory bodies. Pore-like structures indicating pyroptosis are found in DFU tissues, and apoptotic speck protein (ASC)/pyrophosphate oligomer bodies are assembled, thus promoting the development of DFU; the effect of cleaved-caspase-3 on apoptosis was excluded [146]. Besides, the pyroptosis triggered by oxidative stress can also lead to susceptibility to C*andida albicans* in diabetic foot. This evidence indicates that in diabetic foot, some factors may lead to the inhibition of perforin-2 expression, which leads to the susceptibility of diabetes patients. Cells infected by bacteria activate the AIM2 receptor and the marker of pyroptosis, leading to cell death, thus making the wound unable to heal quickly [147]. Another study showed that keratinocytes in a high-glucose environment induced pyroptosis through the NLRP3 pathway, which suggests that pyroptosis may have occurred in some cells of diabetics without infection [148]. The assembled NLRP3 inflammasome can also trigger pyroptosis, activate the protease caspase-1 to induce GSDMD-dependent pyroptosis and facilitate the release of IL-1β and IL-18 [149]. DFU triggers NLRP3 inflammasomes, and partial inhibition of the pyroptosis pathway by blocking NLRP3 may induce DFU. Under stress and inflammatory pathological conditions, active NLRP3 releases caspase-1, which occurs in a cleavage-mediated pyroptosis pattern (Figure 6) [27]. Finally, the research also shows that inhibiting pyroptosis may promote the healing of diabetic wounds [148,150,151]. Pyroptosis can promote the development of diabetic wound/ulcers. Whether the same situation exists in other complications of diabetes mellitus, which links cGAS-STING with pyroptosis, needs further research to verify.

### The cGAS-STING pathway in metabolic reprogramming

**Figure 7:**
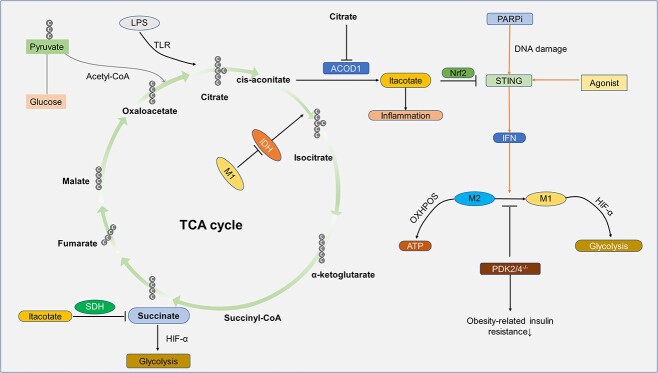
cGAS-STING pathway and metabolic reprogramming. Metabolic reprogramming in macrophages is centered on two TCA cycle breakpoints, namely the *cis*-aconitate withdrawal point when succinate ACDO1 activity is elevated and IDH is blocked, enabling the conversion of *cis*-aconitate into itaconic acid. The antimicrobial properties of itaconic acid and its role as a pro-inflammatory mediator are well-established. It can also inhibit STING activation via Nrf2. Additionally, itaconic acid acts as an inhibitor of succinate dehydrogenase, preventing the conversion of succinate into fumarate. This hinders glycolysis as the primary energy metabolism of M1 via HIF-α, which is less efficient than OXPHOS in M2. STING-IFN activation in M2 leads to its reprogramming to M1, but this process can be inhibited by PDK2/4 deficiency, which also rescues obesity-associated insulin resistance. *LPS* Lipopolysaccharide, *TLR* Toll-like receptor, *CoA* acetyl coenzyme A, *IDH* isocitrate dehydrogenase, *M1* pro-inflammatory macrophages, *ACOD1* aconitate decarboxylase 1, *Nrf2* nuclear factor erythroid 2-related factor 2, *STING* stimulator of interferon genes, *PARPi* PARP inhibitor, IFN interferon, *M2* anti-inflammatory macrophages, *HIF-α* hypoxia-inducible factor -α, *PDK2/4* pyruvate dehydrogenase kinase 2/4, *OXHPOS* oxidative phosphorylation, *SDH* succinate dehydrogenase, *cGAS-STING* cyclic GMP-AMP synthase-stimulator of interferon genes

Metabolic reprogramming refers to the metabolic changes cells make in response to various stimuli. Metabolic reprogramming is common in many diseases involving metabolic pathways such as sugar, lipid and amino acid metabolism, which are closely related to the occurrence and development of diseases. All of these belong to a new discipline—immune metabolism [152–154]. The most familiar of these is the Warburg effect. That is, tumor cells rely on aerobic glycolysis to produce ATP (instead of mitochondrial oxidative phosphorylation, which normal cells rely on). Although the efficiency of ATP production in this way is very low, it gives tumor cells some advantages [153,155]. A vital sign of metabolic reprogramming induced by LPS is the change of metabolites derived from the TCA cycle, especially succinate and itaconate [156]. Succinate plays a pro-inflammatory role, while itaconate plays an anti-inflammatory role (Figure 7) [157,158].

In addition, the polarization of macrophages is another example of reprogramming [159,160]. M1 is mainly powered by glycolysis, while M2 generates ATP by more efficient oxidative phosphorylation (OXHPOS) and participates in tissue repair and anti-inflammation in diabetes. The metabolites of M1 and M2 are also very different [161]. M1 macrophages rely mainly on glycolysis and experience two disruptions in the TCA cycle, leading to the accumulation of itaconate (a fungicide compound), a microbicidal compound and succinate, whereas M2 macrophages depend more on OXPHOS and the TCA cycle is intact [162].

First, aconitate decarboxylase 1 (ACOD1) is upregulated in M1, and *cis*-aconitate can be converted into itaconate by ACOD1 [163], whereas citrate can inhibit ACOD1 activity to reduce the production of itaconate, probably through competitive binding with substrate-binding site [164]. In contrast, IDH is the enzyme that converts isocitric acid to α-ketoglutarate. Subsequent studies have shown that itaconate is able to control its own synthesis by negative feedback inhibition of IDH activity. A break in the metabolism of IDH was identified in M1 macrophages, resulting in a failure of the negative feedback response to clathrate. This provides a mechanistic explanation for the first break in the TCA cycle [163,165]. This leads to a predominance of itaconate production and thus exit of *cis*-aconitate from the TCA cycle [162]. Itaconate can block succinate dehydrogenase, resulting in the inability to convert succinate into fumarate, which is the second break [166]. Reduced itaconate levels improve OXPHOS flux, which may favor M2-like polarization in macrophages. Furthermore, itaconate helps stabilize the levels of the anti-inflammatory transcription factor nuclear factor erythroid 2-related factor 2 (Nrf2) (Figure 7) [162].

Studies have shown that the immune response mediated by cGAS-STING also contributes to cell reprogramming [154,157]. When Toll-like receptor is LPS-induced, the increase of glucose consumption, lactic acid release and TCA cycle in dendritic cells indicate the stimulation of metabolic reprogramming of immune cells. However, STING is time-dependent, which is consistent with the activation trend of Nrf2, suggesting that reprogramming leads to STING being inhibited by Nrf2 [157]. STING agonist can reprogram M2 in tumors to M1 type. At the same time, PARP inhibitor (PARPi) can promote DNA damage and cooperate with M1 to inhibit tumor development [167]. In addition, during infection, an increase in mitochondrial ROS (mROS) mediated by STING is also seen, which can reduce OXPHOS and increase glycolysis by increasing the expression of hypoxia-inducible factor-1α (HIF-1α), and the increase of succinic acid can also lead to an increase in HIF-1α expression, all of which make macrophages reprogram into M1 type to resist the invasion of external viruses or bacteria [168]. In addition to HIF-1α, IFN-1 mediates macrophage reprogramming during this infection, and STING mainly mediates this [169]. It is worth noting that the microorganisms in a high-fiber diet can produce monocyte stimulator Cyclic diadenosine monophosphate (cdAMP), which STING-dependent IFN-1 can reprogram to promote macrophage polarization and improve the immunity and anti-tumor ability of the body [170].

The etiology of DWH, as a metabolic disease, may be strongly related to altered energy metabolism. Macrophage polarization is a good example of this, and in DWH, most evidence suggests that macrophages have an increased glycolytic response, which allows the cells to undergo a metabolic reprogramming reaction with an increase in the metabolite succinate, which leads to an increase in pro-inflammatory M1 polarization. Studies have shown that the simultaneous deletion or inhibition of pyruvate dehydrogenase kinase 2/4 can prevent macrophages from polarizing to M1 phenotype in response to inflammatory stimuli (LPS plus IFN-γ), thus reducing obesity-related insulin resistance [171]. Monocyte activation plays an important role in the development of diabetic complications. Peroxisome proliferator-activated receptor alpha (PPARα) reduces metabolic reprogramming of cells through the cGAS-STING pathway, and PPARα levels are significantly down-regulated in monocytes from diabetic patients and animal models, leading to monocyte activation. This may have great implications for the study of M1 polarization in DWH [172]. There is a strong association between the cGAS-STING pathway and reprogramming, and both may contribute to the development of diabetic complications, especially in the case of macrophage polarization. And modulation of reprogramming through the cGAS-STING pathway to alleviate chronic inflammation in diabetes may be a potential treatment option for diabetic complications (Figure 7).

### The cGAS-STING pathway in cell senescence

**Figure 8:**
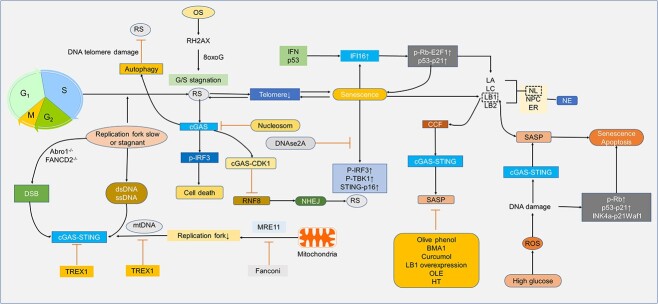
cGAS-STING and cell senescence in diabetes. Cell senescence can be caused by various factors, including oxidative stress, replication fork stagnation, telomere damage and aging. The cGAS-STING pathway plays a crucial role in maintaining genetic stability and preventing tumors by detecting and responding to internal disturbances and external enemies. However, in certain chronic inflammatory conditions such as diabetic high-glucose environments, DNA damage can lead to increased cytoplasmic DNA, activating cGAS-STING and triggering an inflammatory response, ultimately accelerating cellular senescence. Several compounds, including ole, DNase 3 (also called TREX1), HT or curcumol, have been shown to reduce SASP production in aging cells by inhibiting the cGAS-STING pathway. Overall, understanding the role of cGAS-STING in cellular senescence and inflammation can have significant implications for developing new therapies for age-related diseases and chronic inflammatory conditions. *RS* replication stress, *OS* oxidative stress, *rH2AX* a protein of DNA damage response, *8oxoG* 8-oxo-7,8-dihydroguanine, *Rb* retinoblastoma, *cGAS* cyclic GMP-AMP synthase, *cGAMP* 2′3′cyclic GMP–AMP, *STING* stimulator of interferon genes, *TBK1* TANK-binding kinase 1, *IRF* interferon regulatory factor 3, *CDK1* cyclin-dependent kinase 1, *mtDNA* mitochondrial DNA, *TREX1* DNase 3, *DSB* DNA double-strand breaks, *Abro1* Abraxas brother 1, *FANCD2* FA group D2 protein, *dsDNA* double-stranded DNA, *ssDNA* single-stranded DNA, *RNF8* ring finger protein 8, *NHEJ* non-homologous end joining, *IFI16* interferon-inducible 16, *IFN* interferon, *p53* tumor protein P53, *E2F* early 2 factor, *LA* laminin A, *LC* laminin C, *LB1/2* laminin B1/B2, *NL* nuclear envelope includes the nuclear layer, *NPC* nuclear pore complex, *ER* endoplasmic reticulum, *NE* nuclear envelope, *CCF* cytosolic chromatin fragment, *SASP* senescence-associated secretory phenotype, *BMA1* brain and muscle Arnt-like protein-1, *OLE* oleuropein, *HT* hydroxytyrosol, *ROS* reactive oxygen species, *INK4α* p16, *Waf1* p21, *cGAS-STING* cyclic GMP-AMP synthase-stimulator of interferon genes

One symptom of aging cells is cell cycle arrest [173]. During mitosis, nucleosomes compete with cGAS for recognition of chromatin. And at this time cGAS is highly phosphorylated, thus blocking cGAS activation in mitosis. Usually, mitosis lasts only about 30 min, while cGAS signaling takes several hours to complete. Therefore, the phosphorylated IRF3 (p-IRF3) produced during this period cannot activate inflammation. However, in mitotic arrest, the continuous accumulation of p-IRF3 promotes cell death, independent of p53 transcription induction or IFNβ signaling [46,174]. The expression of p-IRF3, p-TBK1 and STING-dependent p16 in aging cells is increased, indicating that activating the cGAS-STING pathway but applying DNase2A degrading enzyme could reduce the innate immune response mediated by cGAS-STING (Figure 8) [175].

Under oxidative stress, rH2AX, dependent on 8-oxo-7,8-dihydroguanine, leads to the stagnation of the replication fork and G1/S phase [176], a sign of replication stress [177]. which subsequently leads to cell senescence [176]. Cell senescence degrades laminin B1 (LB1), increasing CCFs and their escape to the cytoplasmic domain, inducing cGAS-STING activation and SASP expression [11]. The degradation of LB1 is particularly related to aging [11]. As a complex multi-component structure, the nuclear envelope includes the nuclear layer, ER and nuclear pore complex, which are assembled to separate cytoplasm from the nucleus [178,179].

There are four major laminin subtypes in the nuclear layer: laminin A (LA), laminin C (LC), LB1 and LB2 [180,181]. Type B laminin is the primary component of the nuclear layer and it participates in a wide range of nuclear functions, among which LB1 and LB2 are the most representative. They regulate various physiological processes, including cell cycle, proliferation, aging and DNA damage response [182]. They are attached to the inner core membrane and assembled into a discrete fiber web. Studies have shown that the interaction between LA/LC and LB1 is necessary for the normal nuclear fiber layer protein network structure in mouse embryonic fibroblasts [180,181]. Degradation of LB1 in aging cells leads to a change in nuclear shape, but LB1 depletion alone is not enough to lead to aging. LB1 can regulate cell aging when accompanied by cell stress, such as oxidative stress [183]. Inhibition of LB1 leads to cell senescence. Conversely, overexpression of LB1 can slow down cell senescence [182,183]. Activation of p53 and pRB can also reduce the expression of LB1 [182]. In addition, senescent cells experienced degradation of LB1 in the nuclear capsule group and leakage of CCFs, further enhancing the cGAS-STING pathway [11].

Studies have proved that the 293Q mutation of the STING allele, which causes damage to the immune function of cGAS-STING, can weaken the SASP associated with obesity [32]. Similarly, olive phenol can retain the expression of LB1 and partially reduce the expression of SASP [184]. Outside of the brake of the cGAS-STING pathway, brain and muscle Arnt-like protein-1, as a part-time regulator of the long interspersed nuclear element-1 (LINE1)-cGAS-STING pathway, can also inhibit SASP caused by inflammation [185]. Curcumol can inhibit the degradation of LB1 and reduce the release of CCFs and the activation of cGAS-STING, thus reducing the expression of SASP [186]. Oleuropein and hydroxytyrosol can also reduce the expression of SASP induced by cGAS-STING (Figure 8) [184].

In the S-phase process of cell division, the genome under the replication fork is the most unstable [187]. When the replication fork is in a slow or stagnant state, the instability of the replication fork will lead to the triggering of an immune response. At this time, its protective mechanism is fundamental [187]. In the absence of protective components Abraxas brother 1 (Abro1) and FA group D2 protein (FANCD2), it will induce the stagnation of replication fork instability, increase cytoplasmic dsDNA or single-strand-DNA and induce the cGAS-STING immune response [188]. The lack of a protective mechanism may lead to severe DNA DSBs, and similarly, it will also lead to an increase in cytoplasmic DNA, thus inducing cGAS-STING. Unless Trex1 clears the cytoplasmic DNA, the development of an immune response cannot be avoided [188,189]. Lipid overload can also cause replication stress and DNA damage and induce cGAS-STING [190]. Studies have shown that protecting the mitochondrial DNA replication fork is also significant. The Fanconi anemia suppression gene can protect the stability of the mitochondrial replication fork, thus preventing cGAS-STING activation caused by mtDNA produced by meiotic recombination 11 homolog 1 (MRE11) nuclease degradation (Figure 8) [191].

Importantly, cGAS is also a gene stabilizer. cGAS can locate cyclin-dependent kinase at the end of the chromosome, blocking ring finger protein 8. cGAS is also the primary regulator that inhibits mitotic nucleic acid cleavage repair. These processes can block the connection of non-homologous ends of chromosomes, thus inhibiting the repair of DSB and the end-to-end fusion of telomere non-homologous end joining (NHEJ), preventing the occurrence of the replication crisis [192]. cGAS is also the key to starting autophagy after DNA telomere damage. The autophagy defect leads to a replication crisis, which is the beginning of a tumor [193].

cGAS-STING activated after telomere damage will lead to the aggravation of cell aging [194]. Replicative aging cells with reduced telomeres can, in turn, slow down the separation of the replication fork, leading to replication stress [177]. IFI16, a necessary cytoplasmic DNA sensor for the cGAS-STING pathway, is also a senescence marker. Its gene is activated by induction of IFN and p53, and IFI16-α can enhance the expression of p53-p21 and pRb-E2F1 in the cell cycle, thus aggravating cell senescence (Figure 8) [102].

Cell senescence is a critical contributor to diabetes. In the pathology of diabetes, the high glucose microenvironment leads to mitochondrial dysfunction, increasing the ROS and reducing the antioxidant capacity in skin keratinocytes, with mtDNA escaping into the cytoplasm [195]. After massive ROS production [196], the DNA group is attacked and damaged. The oxidized DNA is difficult to decompose, causing accumulation of cytoplasmic abnormal DNA [45], triggering the cGAS-STING pathway and the production of cell cycle inhibitors such as p53-p21 and pRb-p16 ^INK4a^-p21^Waf1^, and increasing senescence and apoptosis [197–199], which enhance the production of various SASPs [200].

In adipose tissues of obese mice and obese humans, STAT1 has been found to work with cGAS-STING to promote growth arrest. STAT3 is a negative STAT1/cGAS-STING signaling regulator that inhibits senescence and inflammation [201]. In addition to adipocytes, this is also true for fibroblasts. A study shows that the reduction of collagen can accelerate cell aging [202]. Cellular oxidative stress increases and leads to cell senescence when the skin is exposed to ultraviolet radiation [203]. Surprisingly, we found that polysaccharides from dendrobium nobile can inhibit the production of oxidative substances in fibroblasts and prevent cell senescence, suggesting another possibility for preventing diabetic complications (Figure 8) [204].

### The cGAS-STING pathway in diabetic complications

#### The cGAS-STING pathway in DN

As a persistent microvascular complication, DN is mainly due to the dysfunction of various renal cells induced by hyperglycemia [205,206], eventually leading to progressive renal failure [207]. The incidence of this disease is increasing year by year, and now it accounts for half of end-stage renal disease [205]. The kidney is the second largest oxygen-consuming organ in the body, so it is susceptible to the energy metabolism of mitochondria. Previous studies have shown that hyperglycemia damages renal tubular cells and causes metabolic disorders of cells [208].

**Figure 9:**
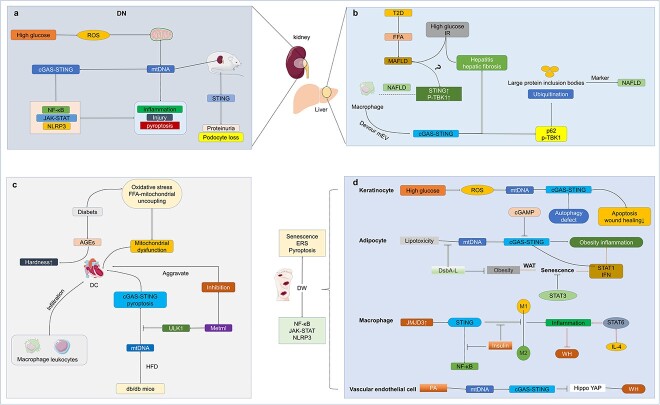
The role of cGAS-STING pathway in diabetic complications. (**a**) In DN, the high glucose environment generates large amounts of ROS, which disrupts mitochondrial function and leaks mtDNA to the extracellular compartment, which activates the cGAS-STING pathway and leads to the activation of the downstream NF-κB, JAK-STAT, and NLRP3 inflammatory pathways, resulting in kidney injury. (**b**) MAFLD belongs to a class of NAFLD caused by excessive FFA accumulation in T2D, MAFLD leads to the development of IR and progression to hepatitis and cirrhosis. In NAFLD, macrophages are able to activate cGAS-STING via EVs and later phosphorylate p62 and TBK1, producing lipotoxicity-induced ubiquitination and large protein inclusions, which are hallmarks of NAFLD, leading to hepatitis and cirrhosis development. (**c**) AGEs are increased in diabetic myocardium, which can lead to myocardial sclerosis. Oxidative stress is also increased in diabetic myocardium and FFA-associated mitochondrial uncoupling occurs, which all contribute to the development of DC. At the same time, leukocytes such as macrophages begin to activate. At the mechanistic level, *db/db* mice are fed via HFD, which leads to mtDNA leakage and activation of cGAS-STING as well as the pyroptosis pathway, leading to the development of DC. In contrast, Metrnl was able to inhibit cGAS-STING as well as pyroptosis via ULK1, thereby alleviating DC. (**d**) In diabetes skin tissues and adipocytes, the high glucose microenvironment will lead to the excessive production of ROS in keratinocytes, destroy mitochondrial function, and lead to the release of mitochondrial DNA into the cytoplasm, thus activating the cGAS-STING pathway, which leads to increased apoptosis, thus inhibiting DWH. Lipotoxicity in adipocytes can also lead to the release of mtDNA, thereby activating the cGAS-STING pathway and leading to obesity type inflammation. DsbA-L can prevent the occurrence of this pathway, thereby inhibiting the aging caused by obesity. STAT1/3 plays two opposite roles in this process. STAT3 can inhibit obesity induced aging, while STAT1 and IFN can promote inflammation caused by cGAS-STING in obesity. In macrophages, JMJD3 causes STING activation and increases M1 polarization, resulting in delayed DWH, which can be inhibited by insulin. PA can also lead to the release of mtDNA from vascular endothelial cells and the activation of cGAS-STING, which can inhibit the HIPPO-YAP classic pathway, thereby inhibiting angiogenesis and slowing down DWH.* JMJD3 *jumonji domain-containing protein-3, *DN* Diabetic nephropathy, *ROS* reactive oxygen species, *mtDNA* mitochondrial DNA, *cGAS* cyclic GMP-AMP synthase, *cGAMP* 2′3′cyclic GMP–AMP, *STING* stimulator of interferon genes, *TBK1* TANK-binding kinase 1, *IRF3* interferon regulatory factor 3, *T2D* type 2 diabetes, *FFA* free fatty acid, *MAFLD* metabolic dysfunction-associated fatty liver disease, *IR* insulin resistance, *NAFLD* non-alcoholic fatty liver disease, *AGEs* advanced glycation end products, *DC* diabetic cardiomyopathy, *ULK1* UNC-52-like kinase 1, *HFD* high-fat diet, *ERS* endoplasmic reticulum stress, *DsbA-L* disulfide bond A oxidoreductase-like protein, *WAT* white adipose tissue, WH wound healing, *PA* palmitic acid, *cGAS-STING* cyclic GMP-AMP synthase-stimulator of interferon genes

As mentioned above, high glucose can lead to an increase in ROS in cells. The increase in ROS and the autophagy defect of mitochondria in DN patients may lead to the dysfunction of mitochondria in the kidney and an increase in mtDNA damage [208–210]. High glucose can lead to the damaging of intracellular mtDNA and an increase in extracellular release, so mtDNA is generally regarded as a potential biomarker of DN [210–212]. mtDNA may be excreted mainly through renal filtration because, in diabetic patients, mtDNA is less expressed in plasma and mainly concentrated in the urine (Figure 9a) [211].

Similarly, in mice with an overload of mtDNA, the content of mtDNA in urine increased, which led to kidney inflammation and injury, indicating that mtDNA may be involved in the pathological mechanism of DN [213]. Other studies also showed that the inflammatory pathway of NF-κB, JAK–STAT, NLRP3 and pyroptosis was found in DN [214–221], and these inflammatory pathways could be expressed downstream of cGAS-STING. Therefore, it is speculated that cGAS-STING may be involved in the development of DN [212]. In subsequent experiments, it was found that the expression of p-STING, p-TBK1 and p-IRF3 in the kidneys of DN mice increased, proving that the cGAS-STING pathway was activated, and mtDNA produced by damaged mitochondria was probably responsible for that activation (Figure 9a) [24].

As predicted by Rayego-Mateos and others, mtDNA does participate in the pathological changes in DN mice [204] but it is not known whether it is the main factor [22]. Studies have shown that STING is activated in diabetic mice while activating STING in wild-type mice with specific agonists leads to proteinuria and podocyte loss [23]. Podocyte loss is an inflammatory nerve injury disease, a unified potential marker of DN [222,223]. In the DN of mice, STING activation is first found in the glomerular position, and STING inhibition can delay the development of DN and prolong the life of mice [23]. This also implies that STING may be one of the main reasons for the development of DN (Figure 9a).

Another study also proved that cGAS-STING was activated in DN and promoted the damage of DN podocytes, which was caused by activation of the NF-κB pathway downstream of cGAS-STING, but not IRF3, one reason being that IRF3 phosphorylation or IFN-β levels remain unchanged in DN mice. Therefore, it is the use of STING or TBK1 inhibition or knockdown that can slow down the progression of DN and damage to podocytes [224]. However, inhibiting the opening of mPTP does not inhibit the activation of cGAS-STING, but the activation of cGAS-STING is not limited to mtDNA, so this point needs further study [224]. The development of cGAS-STING and DN may be closely related to mtDNA damage. cGAS-STING may act differently in different cells and cannot be generalized (Figure 9a).

#### The cGAS-STING pathway in diabetic hepatopathy

Non-alcoholic fatty liver disease (NAFLD) has become an epidemic with an increased prevalence of obesity and T2D, most likely contributing to the increase in NAFLD patients [225]. T2D-induced NAFLD is known as metabolic dysfunction-associated fatty liver disease (MAFLD) and is considered to occur in all types of diabetes mellitus and in individuals with biomarkers, imaging or biopsy findings that include >5% liver fat [226]. T2D complicated with liver disease is generally divided into several stages, from free fatty acid [palmitic acid (PA)] accumulation into the fatty liver to hepatitis, and then develops into liver cirrhosis, and finally evolves into liver cancer [227–229]. According to one study, there is a 5-fold higher prevalence of NAFLD in patients with T2D compared to that in patients without T2D [228]. Furthermore, hyperglycemia and insulin resistance are involved in the development of fatty liver, hepatitis and liver fibrosis [227–230].

Analysis of liver samples from patients with NAFLD showed increased expression of STING/p-TBK1 in all cells compared to the normal control group, and predominantly in macrophages, especially in the porta hepatis of patients with Non-alcohol-associated fatty liver (NASH) fibrosis compared to the healthy control group [231,232]. Vsig4+ macrophages can remove intestinal microbial DNA-containing extracellular vesicles (mEVs) from the blood through a C3-dependent conditioning mechanism. Its reduction leads to a greater vulnerability of hepatocytes to the accumulation of intestinal microbial DNA exosomes and the initiation of hepatitis by cGAS-STING [233]. These results indicate that cGAS-STING is activated in patients with NAFLD and that macrophage STING may serve as a new therapeutic target for NAFLD [234].

In another study, it has been found that fatty liver and NAFLD in mice are alleviated after the knock-out or inhibition of STING [130,235,236]. There is evidence that hepatocytes do not express STING, which leads to adaptive replication of hepatitis virus [237], and further analysis is needed to clarify this gap. Besides, the activation of cGAS-STING, followed by p-TBK1 phosphorylation of p62, contributed to hepato-lipotoxicity-induced ubiquitination and large protein inclusion bodies, which are markers of non-alcoholic steatohepatitis [238]. Much evidence has shown that cGAS-STING is involved in the development of NAFLD. However, it is still not known whether cGAS-STING occurs in MAFLD, so in the future it can be tested whether the cGAS-STING axis is activated and its effect on the development of MAFLD in diabetic liver disease can be investigated (Figure 9b).

#### The cGAS-STING pathway in diabetic cardiomyopathy

Diabetic cardiomyopathy (DC) was initially described as a human pathophysiological condition in which heart failure occurs without coronary artery disease, hypertension and valvular heart disease [239,240]. The prevalence of DC increases with the number of diabetic patients. Mitochondrial dysfunction, increased oxidative stress, AGEs, inflammation and microvascular dysfunction are all related to the development of DC [241,242]. The increased formation of AGEs may change structural proteins and lead to increased myocardial hardness [243]. The infiltration of macrophages and leukocytes and the increased expression of inflammatory factors lead to the myocardial inflammatory reaction [244–246]. Inhibition of inflammatory reaction can alleviate or prevent DC [247,248]. In addition, oxidative stress and ultrastructural abnormalities were observed to occur in the mitochondria of diabetic myocardial cells, and fatty acids also increased mitochondrial uncoupling, leading to mitochondrial dysfunction [249–251].

Studies have shown that the increase of cytoplasmic mtDNA in *db/db* mice fed with HFD and the activation of cGAS-STING signaling pathways and downstream targets such as IRF3 and NF-κB may be due to the increase of ROS and the impairment of mitochondrial function in myocardial cells of diabetic mice due to lipotoxicity. The activation of the cGAS-STING inflammatory pathway is also induced by cytoplasmic mtDNA, thus accelerating the development of diabetic cardiomyopathy [25]. Adeno-associated virus knocks down the expression of STING in diabetic mice. Then PA-induced lipotoxicity produces excessive ROS, which damages the mitochondria of myocardial cells and leads to the release of a large amount of mtDNA into the cytoplasm, which activates the cGAS-STING pathway of myocardial cells and its downstream pyroptosis, thus worsening the development of DC [28].

It is noteworthy that the expression of meteor-like hormone (Metrnl) is decreased in diabetic cardiomyopathy. Metrnl can activate the autophagy pathway and dephosphorylate STING through phosphorylation of ULK1, thus inhibiting cGAS-STING signal transduction in myocardial cells. Down-regulating the expression of Metrnl will aggravate the damage of myocardial cells under high glucose, while over-expressing the protein can alleviate the development of DC [33]. Finally, although there are few studies on cGAS-STING in DC, it can be seen that knocking out STING or overexpressing Metrnl can alleviate the heart injury and dysfunction of diabetic mice and inhibit the inflammatory state of myocardial cells. However, we need to weigh the advantages and disadvantages against the harm caused by the suppression of STING. In the future, more evidence is needed to prove the deep mechanism of cGAS-STING in DC (Figure 9c).

#### The relationship between cGAS-STING and DWH

T2D, the most common type of diabetes, is characterized by insulin resistance due to overnutrition and innate immune activation [15,16]. One of the diabetes complications, DWH, is one of the most common, severe and costly to treat. According to IFD statistics, every 30 s, a diabetic patient loses a lower limb or part of the lower limb due to diabetes. The healing process of skin injury can be divided into hemostasis, inflammation, proliferation and remodeling [252–256]. In the inflammation stage, an increase in inflammatory cells and inflammatory factors can be observed [256,257].

Chronic inflammation is a significant feature of DWH [19], in which the polarization of macrophages is a crucial step in the inflammatory stage of wound healing [258–260]. Studies have found that pro-inflammatory macrophages (M1) in DWH are difficult to polarize into anti-inflammatory macrophages (M2) [260,261]. M2-type macrophages have a great benefit in wound healing, e.g. puerarin can significantly inhibit the NF-κB and mitogen-activated protein kinase signaling pathways, down-regulate the expression of inflammatory cytokines and induce macrophage M2 polarization, which leads to the improvement of DWH [262].

In a recent study on DWH, researchers discovered that STING is highly active in the wounds of diabetic patients. This heightened STING activity seems to fuel persistent inflammation in these wounds. Interestingly, this overactivity is most pronounced in the later stages of wound healing and appears to be a major factor in diabetic wound formation. High glucose levels, common in diabetes, and STING activators worsen inflammation, slowing down healing. Furthermore, high glucose triggers STING through a process involving cellular damage. STING inhibitors speed up wound healing by calming down this inflammation, while STING activators make it worse. When researchers blocked STING in certain immune cells, the wound environment was shifted towards a more healing-friendly state. These findings suggest that targeting STING might be a way to improve DWH [263].

It is interesting to note that the expression of STING and inflammatory factors in macrophages of diabetic wounds increased [26]. Besides macrophages, the activation of cGAS-STING can also be identified in non-immune cells such as mouse fibroblasts and adipocytes [195,264,265]. Studies have shown that signal transduction pathways, such as NF-κB, JAK–STAT3 and NLRP3 inflammatory corpuscles, all contribute to the development of DWH [27,266,267]. Cell senescence, ERS and focal death were also detected in DWH, contributing to chronic inflammation [268,269]. However, the details of how these signals or pathways cross-talk with cGAS-STING in DWH are still unclear. Here, we will briefly introduce the role of cGAS-STING in DWH and extend the introduction to obese chronic skin inflammation. cGAS-STING is mainly related to skin diseases. However, the effect of cGAS-STING on different skin cells is not the same (Figure 9d).

Activation of the cGAS-STING pathway can cause inflammation of keratinocytes, which plays a vital role in diabetic skin inflammation. It was found that ROS production of keratinocytes was increased in a high-glucose environment, and the accumulation of ROS led to the dysfunction of mitochondria and an increase of mtDNA fragments in the cytoplasm, which led to the activation of the cGAS-STING pathway [195]. Similarly, the expression of STING was also increased in *db/db* mice and keratinocytes treated with high glucose, which may be related to the autophagy function defect induced by high glucose [270]. In these cases, the cGAS-STING inflammatory pathway of keratinocytes was activated, the apoptosis of cells increased, and finally, wound healing was delayed. Nevertheless, the STING-IRF3 pathway of HaCaT cells was also activated in DWH patients with psoriasis [271,272]. However, the co-localization of IFI16 with STING during this procedure was critical for recruiting TBK1 (Figure 9d; Table 1) [273].

**Table 1:** Role of cGAS-STING pathways in diabetic wound healing

**Inducing factors**	**Relationship**	**Cell**	**Reference**
The epidermis of STZ-induced diabetes in mouse and *db/db* mouse models.	STING was activated in the epidermis of diabetic mice, and the STING signal was increased by autophagy disorder, which delayed wound healing. Inhibiting STING accelerates wound healing.	HaCaT	[270]
Palmitic acid (PA)	PA treatment induced mitochondrial DNA (mtDNA) to release into cytosol and activated the cGAS-STING-IRF3 signal of the cytoplasmic DNA sensor. The activated IRF3 binds to the MST1 gene promoter and induces MST1 expression, resulting in MST1 up-regulation, yes-associated protein (YAP) inactivation and angiogenesis inhibition.	Endothelial cells	[289]
JMJD3	IL-6 produced in the late stage of diabetes can regulate the JMJD3 of macrophages through JAK1,3/STAT3 signals, thus inducing inflammatory pathways such as NF-κB, and STING is regulated by it. Inhibiting the expression of JMJD3 can reduce the expression of inflammatory factors, thus speeding up wound healing.	Macrophages	[26]
Psoriasis with diabetes mellitus	STING-IRF3 pathway was activated in diabetic mice with human immortalized keratinocytes (HaCaT) treated with PA and imiquimod (IMQ) or psoriasis induced by IMQ. STING inhibitor C176 can improve the inflammatory state of dermatophytes in diabetes.	HaCaT	[271]

*cGAS-STING* cyclic GMP-AMP synthase-stimulator of interferon genes

Lipotoxicity may be one of the masterminds leading to chronic inflammation of diabetic wounds [274,275], Brown adipose tissue has a high density of mitochondria, and its main function is to generate heat, so it is sensitive to changes in mitochondria [264]. Several lines of evidence have pointed out that obesity leads to the aging of white adipose tissue cells. Aging leads to the activation of IRF and STAT1/3 signals. STAT3 is a negative regulator, while STAT1 leads to growth stagnation and activates cGAS-STING6 [201]. In adipose tissue of insulin-resistant mice, lipotoxicity, such as that induced by PA, induces mitochondrial damage in adipocytes and increases cytoplasmic mtDNA, thus activating the cGAS-STING pathway (Figure 9d) [276].

Activating cGAS-STING leads to insulin resistance and inhibition of thermogenesis and has a crucial effect on lipid metabolism [234,264,277]. In contrast, disulfide bond A oxidoreductase-like protein (DsbA-L) in adipocytes can reduce the release of mtDNA and protect mice from obesity induced by a HFD [15,264]. Interestingly, however, in obese mice, topical cGAMP can show an anti-inflammatory reaction in the liver and adipose tissue induced by HFD and reduce the hypoglycemic effect of glucagon and gluconeogenesis [278]. This suggests that cGAMP may activate the negative feedback system of the cGAS-STING pathway, thus reducing obesity-induced chronic inflammation. Of great interest, a lipid-enriched diet can improve the reactivity of endogenous retrovirus in the skin, leading to increased immune response and tissue inflammation.

The endogenous virosome of the skin is mainly used to communicate with the external microbiota, making an essential contribution to the homeostasis of the inflammatory response in the tissue [279]. Unfortunately, there is no research on the role of the cGAS-STING pathway in adipocytes of diabetic wounds, which may be due to too much interference in detecting subcutaneous adipocytes. However, we can obtain relevant information from the epididymal adipose tissue of most obesity models, providing a new perspective for future research into diabetic skin injury (Figure 9d; Table 1).

The failure of M1-type macrophages to convert to M2-type in diabetic wounds contributes to the continuous inflammation of DWH [26,280,281]. Previous work has identified the gene transcription profiles of normal and diabetic wound macrophages. A change is identified in pro-inflammatory and pro-fibrotic gene expression, such as IFN-γ, the central inflammation regulators [282,283]. In addition, upregulation of the STING gene has been identified in single-cell sequencing [283]. It was found also that the expression of JMJD3 in diabetic wound macrophages increased, and it positively regulated the activation of STING in macrophages. Moreover, inhibiting the expression of JMJD3 can also inhibit the activation of STING (Figure 9d) [26].

Furthermore, in diabetic cutaneous wound, various inflammatory signaling pathways are activated and cross-talk, which is also closely related to innate immune cells [27]. Studies have shown that NF-κB of macrophages in diabetic skin injury is up-regulated, which may be partly caused by the activation downstream of cGAS-STING [284–286]. As found in the research, STING can degrade IκB by activating IκK, thus canceling the restriction of IκB on NF-κB and causing the dimerization and activation of NF-κB [45]. Moreover, the application of insulin can promote the polarization of macrophages and inhibit the expression of NF-κB, thereby reducing inflammation [266]. The regulation of the JAK–STAT signaling pathway can also significantly affect the skin wound healing of diabetes. When IL-4 stimulated diabetic wounds in mice, the phosphorylation of STAT6 was enhanced, and the proportion of M2 macrophages decreased, resulting in delayed wound healing (Table 1) [287].

Angiogenesis also involves the cGAS-STING pathway. PA is a major free fatty acid (FFA) in metabolic syndrome, accounting for 27% of the total FFA in plasma [288]. PA can induce the damage of mtDNA, activate the cGAS-STING signal pathway in endothelial cells and destroy the Hippo-YAP pathway, thus inhibiting angiogenesis and wound healing, which were associated with increased expression of mammalian Ste20-like kinases 1 (MST1), YAP phosphorylation/inactivation and nuclear exclusion [289]. In a word, chronic inflammation appears in diabetic or obese skin, which leads to delayed wound healing, and this is related to the activation of the cGAS-STING pathway of cells in the skin. Although few studies are available, with the current state of research described above, we suggest that appropriate inhibition of the cGAS-STING pathway may reduce the inflammatory state of the skin and accelerate wound healing (Figure 9d; Table 1).

### Novel therapies emerging in disease to target the cGAS-STING pathway

#### Agonists

RocA, a natural product, can also increase the expression of C-C chemokine ligand 5 by targeting mtDNA to activate cGAS-STING signal transduction, thus promoting the infiltration of NK cells [290,291]. In addition, the binding capacity of cGAS to DNA can be enhanced and the cGAS-STING signal can also be enhanced. β-Arrestin 2 is a compound that directly interacts with cGAS, which enhances the binding of dsDNA and the production of cGAMP through deacetylation at position 171. Lys171 is the critical residue of β-blockin2, and its deacetylation is necessary for the activation of cGAS (Table 2) [292–299].

**Table 2:** Regulation of the cGAS-STING pathway

**Agonist**		
**Compound**	**Molecular mechanism**	**Reference**
Enhancer of zeste homolog 2 (EZH2)	The EZH2-HMGA1-USP7 complex regulates the formation of CCF, and CCF activates cGAS, but USP7 is required to de-ubiquitinate cGAS and stabilize cGAS, EZH2 can promote breast cancer metastasis through cGAS-STING pathway activated by CCF.	[293]
Dispiro diketopiperazines (DSDP)	DSDP, a Human STING agonist, can induce an interferon-dominant cytokine response in human skin fibroblasts and peripheral blood mononuclear cells. It potently suppressed the replication of yellow fever, dengue and Zika.	[294]
ADU S-100	Stimulating STING to promote an immune response within The tumor microenvironment (TME), a low dose of ADU S-100 can lead to the infiltration of immune cells in TME, slowing down the growth of melanoma and decreasing angiogenesis.	[295]
Cyclic di-GMP (CDG)^SF^	CDG excites STING and enhances the immune response, which can be used in cancer treatment and as an adjuvant to enhance the efficacy of the SARS-CoV-2 vaccine.	[296]
**Inhibition**		
**Compound**	**Molecular mechanism**	**Reference**
MRT67307	The inhibition of TBK1-IRF3-IFNβ blocks UVB-induced apoptosis of HaCaT cells.	[8]
C-176	The inflammatory responses in the skin tissue of diabetic mice with psoriasis were ameliorated by treatment with C-176.	[271]
	Silencing of STING accelerated wound healing *in vitro*. *In vivo*, it inhibits the inflammatory response in epidermis and accelerates wound healing in diabetic skin.	[270]
	Blocked the nuclear translocation of P-IRF3 and NF-κB P65 and alleviated the kidney injury induced by Trichloroethylene (TCE) sensitization.	[297]
H151	The inhibition of STING reduces SARS-CoV-2-induced inflammation in mice.	[298]
	8-Oxoguanine DNA glycosylase (OGG1) regulates IFN-β expression through the cGAS-STING pathway, use of the STING inhibitor, H151, reduced both basal and cGAMP-driven increases.	[299]
**Metal ions**		
**Metal**	**Molecular mechanism**	**Reference**
Zn^2+^	Zn^2+^ increases cGAS activation by promoting the phase separation of the cGAS-DNA complex. Zn^2+^ can produce ROS and inhibit autophagy, significantly enhancing cGAS-STING signaling. In addition, Zn^2+^ also makes laminin assemble, destroys the stability of the nuclear structure, and induces apoptosis. TPEN is a Zn^2+^/Fe^2+^ chelating agent that can prevent this process from happening.	[327,330,331]
Mn^2+^	Mn^2+^ could promote the escape of mtDNA and enhance the affinity of STING and cGAMP. Mn^2+^ was included in different preparations to enhance immunity, serving as a delivery system to stimulate humoral and cellular immune responses.	[328,332]
Ca^2+^	Ca^2+^ regulates the connection between STIM1 and STING, STIM1 inhibits the transport of STING. The overload of Ca^2+^ in mitochondria will lead to the opening of mPTP channel and the leakage of mtDNA. Ca^2+^ overload will open the mPTP channel and may lead to the activation of STING.	[335–337]
Fe^2+^	Fe^2+^ activates cGAS-STING signaling and promotes hepatic inflammation, which promotes liver inflammation.	[338]

*cGAS-STING* cyclic GMP-AMP synthase-stimulator of interferon genes, *USP* ubiquitin-specific peptidase, *CCF* cytosolic chromatin fragment, *TME* tumor microenvironment, *CDG* cyclic di-GMP, *IRF* interferon regulatory factor, *IFN* interferon, *TCE* Trichloroethylene, *OGG1* 8-Oxoguanine DNA glycosylase, *STIM1* stromal interaction molecule 1

However, inhibiting DNA repair can have a similar effect. For example, SRC homology-2 domain-containing protein tyrosine phosphatase-2 inhibits DNA repair mediated by PARP1, strengthening the cGAS-STING pathway [300]. high-grade gliomas (HGGS) encodes Gly-34 Arg/Val substituents in histone H3.3 (H3.3-G34R/V) that plays a role in stabilizing genes. The application of DNA damage response inhibitor-pamiparib/AZD1 can prolong the life-span of mice carrying H2.7762-G50R pHGG, which may play a role by activating cGAS-STING (Table 2) [301].

Cyclic dinucleotides (CDNs), a collection of cyclic dinucleotides and a specific activating ligand for STING, are composed of cyclic di-GMP, cyclic di-AMP and cGAMP. Furthermore, human STING activates 2'5'-cGAMP [127,302–314]. In addition to the traditional CDNs, ADU-V19, ADU-S100, diABZI STING agonist-1 (tautomerism), DMXAA, IACS-8779, IACS-8803, E7766, BMS-986301. IMSA101, MK-1454, SB11285, SR-717, PC7A and MSA-2 are all agonists of STING. Further, better efficacy can be obtained when STING agonists are coupled to anti-tumor antibodies to form antibody–drug conjugates [315]. Bortezomib can also activate cGAS-STING and induce apoptosis of multiple myeloma cells [316]. Compounds C-23, C-26 and C-27 designed according to C-170 inhibitors are likely to become activators of STING [304]. Osimertinib can also activate cGAS in cancer cells to produce cGAMP, which can trans-activate STING in macrophages (Table 2) [317].

#### Inhibitors

On the other hand, blocking any link of the cGAS-STING pathway may reduce inflammation. First, we can stop the release of DNA. For example, PPARα may regulate cGAS-STING signal transduction through mtDNA release. Therefore, regulating the PPARP pathway may regulate the activation of cGAS [318]. Moreover, tanreqing has been proven to inhibit mtDNA release and STING-mediated signal pathways *in vitro* [311]. Secondly, inhibiting the recognition of cGAS can also inhibit the occurrence of inflammation. RU.521, as an inhibitor of cGAS, can reduce IFN and focal death induced downstream of STING [25,304,305]. Finally, blocking the activation of STING or its downstream pathway may also slow down the occurrence of inflammation. STING-specific inhibitors C-170 and C-176 can inhibit the combination of cGAMP and STING [25,304]. Genistein can block the interaction between STING and TBK1 and IKKε and directly block the activation of the innate immune response downstream of STING [319].

H-151 can also inhibit STING/NF-κB signaling in keratinocytes and immune cells [320], and the use of amlexanox in pulpitis can inhibit the activation of TBK1 [321]. cGAS is also inhibited by receptor tyrosine kinase HER2, which directly recruited AKT1 to phosphorylate the S510 site of TBK1, breaking the ubiquitination connection of STING and TBK1, thus blocking the downstream immune response [322]. Regardless, after the conformational change of STING, the binding force of sulfated glycosaminoglycans to STING in the Golgi apparatus may be enhanced, resulting in decreased binding of STING to TBK1, thereby blocking the inflammatory pathway [323]. Similarly, mutated p53 (mtp53) protein binds to TBK1, which prevents the aggregation and activation of TBK1 with STING and IRF3, leading to a decrease in the immune response (Table 2) [324].

In addition, the application of insulin can promote the polarization of macrophages and inhibit the expression of NF-κB, thereby reducing inflammation [266]. Laser at 660 nm can also regulate the gene expression of the JAK–STAT signaling pathway in diabetic wound cells, which also promotes wound healing [325]. Tofacitinib and aspirin can inhibit JAK–STAT and NF-κB signaling pathways, thus inhibiting the development of T2D [326]. These cases play a role by inhibiting the cGAS-STING pathway, but whether they can all be applied to diabetic complications needs further exploration (Table 2).

#### Metal ions

As critical components or modulators of many enzymes, metal ions can promote or inhibit the progression of cGAS-STING and thus exhibit the function of regulating inflammation. It has been reported that Zn^2+^ increases cGAS activation by promoting the phase separation of the cGAS–DNA complex [327,328]. TPEN, a Zn^2+^ chelating agent, can inhibit cGAS activation in cells, but TPEN can also chelate Fe^2+^ and Mn^2+^ and some other divalent cations [327,329]. By forming nanoclusters of ZnS@BSA (bovine serum albumin), Zn^2+^ showed a significant enhancement of the cGAS-STING signal, and intracellular Zn^2+^ was able to generate ROS [330]. The latest research has found that the preparation of Zn^2+^ adjuvant could block autophagy, induce mitochondrial damage and activate cGAS-STING, thus effectively enhancing the tissue immune response (Table 2) [331].

In addition, there was evidence that Mn^2+^ could promote the escape of mtDNA and other disorders in the steady growth state of cells [328] and further enhance the affinity of STING and cGAMP, thereby making STING more easily activated [332]. This is indispensable for resisting virus invasion through cGAS-STING [328,332]. Mn^2+^ was included in different preparations to enhance immunity, serving as a delivery system to stimulate humoral and cellular immune responses [333,334]. As mentioned above, excessive ROS can cause mitochondrial disorder and thus produce mtDNA escape, an intermediate process that has been proved in macrophages. ROS oxidizes DNA to become Ox-DNA, while unrepaired ox-DNA is cleaved into 500–650 bp fragments by endonuclease FEN1. Then overloading of Ca^2+^ in the mitochondria leads to the opening of the mPTP channel through which these fragments leave the mitochondria to initiate cytoplasmic NLRP3 inflammasome activation and cGAS-STING signaling (Table 2) [335].

**Figure 10:**
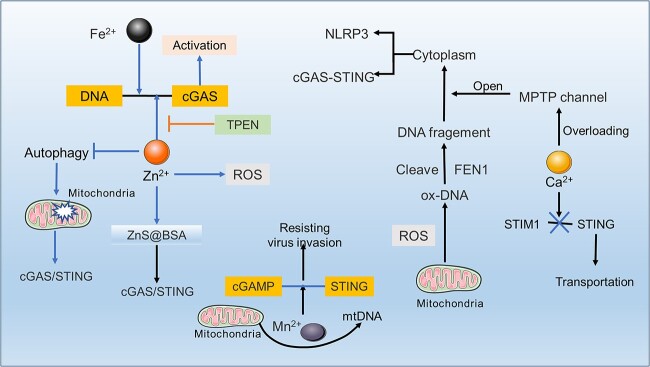
Regulation of cGAS-STING by metal ions. Both Fe^2+^ and Zn^2+^ increase the binding affinity of cGAS to DNA, thereby activating the cGAS-STING pathway. However, the action of Zn^2+^ can be inhibited by the Zn^2+^ chelating agent TPEN. Additionally, Zn^2+^ can increase the production of reactive oxygen species and inhibit autophagy, leading to mitochondrial dysfunction and subsequent activation of the cGAS-STING pathway. The ZnS@BSA (bovine serum albumin) preparation of Zn^2+^ can also activate the cGAS-STING pathway. Similarly, Mn^2+^ can enhance the signaling of cGAS-STING by increasing the binding affinity between cGAMP and STING, promoting the release of mtDNA. Furthermore, excessive Ca^2+^ can cause the opening of the mitochondrial permeability transition pore, allowing mtDNA to escape and activate both the cGAS-STING pathway and NLRP3 inflammasomes. In the endoplasmic reticulum, Ca^2+^ mediates the transport of STING, blocking the localization of STING, leading to its transportation to the ERGIC and Golgi for activation. *cGAS* Cyclic GMP-AMP synthase, *cGAMP* 2′3′cyclic GMP–AMP, *STING* stimulator of interferon genes, *TBK1* TANK-binding kinase 1, *IRF3* interferon regulatory factor 3, *BSA* bovine serum albumin, *TPEN* a chelator with strong affinities for Zn^2+^, Fe^2+^ and Mn^2+^, *mtDNA* mitochondrial DNA, *oxDNA* oxidative DNA, *FEN1* flap endonuclease 1, STIM1 STING and protein-matrix interacting molecule 1

Furthermore, Ca^2+^ also regulates the transport of STING, displaying the ability to control STIM1, the sensor matrix interaction molecule of STING, thereby limiting the transport of STING from ER to the Golgi apparatus. |A lack of STIM1 leads to the enhanced activation of STING [336]. Ca^2+^ overload will inhibit the accumulation of STIM1, which is the body’s protection mechanism [337]. More interestingly, cGAS also appears to be activated by overloaded Fe^2+^ (Figure 10) [338]. In conclusion, metal ions are essential in regulating the cGAS-STING pathway. Therefore, in the future, some metal-ion chelating agents may be designed to treat more inflammatory diseases related to cGAS-STING.

### Treatment of diabetic wounds and other inflammatory diseases by mediating the cGAS-STING pathway

In diabetes, the inhibition of STING expression can promote wound healing [270]. In the case of skin injury, external bacteria or viruses invade the interior of the wounded tissue and produce large amounts of heterologous cDNA. Afterward, cGAS dimerization is activated [1]. Mizutani Y *et al*. treated mouse skin trauma with an ointment containing cGAMP, increasing type I IFN through the TBK1-IRF3 pathway and accelerating the healing of common skin wounds but not DWH [339].

Several small-molecule inhibitors targeting cGAS or STING have recently been developed and show promise for dampening inflammation in diabetes and DWH. For example, the STING inhibitor C-176 can reduce NF-κB activation and proinflammatory responses *in vivo*, accelerating wound healing in diabetic mice [23,263]. Other inhibitors like H-151 also mitigate STING-mediated inflammation and injury in animal models of DC [25,224,270,340,341]. In addition to synthetic inhibitors, some endogenous factors like PPARα and Metrnl act as natural repressors of STING signaling [33,172]. On the other hand, STING agonists such as cGAMP and cyclic diAMP exacerbate inflammation and complications like DN [23,263]. The differential effects of cGAMP in type 1 versus type 2 diabetes models underscores the need for further research [329,342,343]. While inhibition of overactive cGAS-STING signaling shows potential for treating diabetic wounds, optimal dosing and timing requires further study to prevent immunosuppression [23,263]. Development of topical inhibitors represents an attractive approach to target hyperinflammation locally in wounds [278]. More specific inhibitors of downstream signaling like JAK-STAT may also enable tuning of cGAS-STING activity [26]. Overall, pharmacological modulation of cGAS-STING is a promising yet underexploited therapeutic strategy for DWH that warrants expanded research.

### Perspectives

The cGAS-STING pathway presents promising new opportunities to understand and treat impaired healing in diabetic wounds. Aberrant cGAS-STING signaling contributes to sustained inflammation, a key factor underlying delayed wound repair. However, many open questions remain regarding the specific mechanisms connecting cGAS-STING to defective healing. Further research to elucidate these interactions can unveil novel therapeutic targets within this pathway. Early findings suggest that inhibiting cGAS-STING to dampen inflammation may improve healing outcomes. But optimized timing and dosing of treatment requires additional investigation to balance potential risks. Future studies should also explore the impact of modulating cGAS-STING in other understudied cell types involved in wound healing. Another frontier is determining relationships between cGAS-STING signaling and pathways like oxidative stress, cellular senescence and metabolic dysfunction that are dysregulated in diabetic wounds. A better understanding of these interconnected mechanisms will expand strategies to correct the chronic inflammatory state obstructing proper healing.

## Conclusions

In conclusion, the cGAS-STING pathway has emerged as a key driver of inflammation and defective healing in diabetic wounds. Aberrant activation of the cGAS-STING cascade triggers proinflammatory signaling and disrupts cellular processes critical for proper wound repair. However, many questions remain regarding how exactly cGAS-STING signaling connects to other pathways implicated in impaired DWH, like inflammation, cellular senescence and metabolic dysfunction. Elucidating these mechanisms is crucial to exploiting modulation of the cGAS-STING pathway as a therapeutic strategy. While early findings support targeting cGAS-STING to reduce inflammation and improve healing, further research is needed to translate these approaches into viable clinical treatments. Overall, the cGAS-STING pathway represents an underexplored target with significant potential for improving outcomes in patients with diabetic wounds. Further studies focused on cGAS-STING mechanisms and therapeutic inhibition can unveil new directions to address this serious complication of diabetes.

## Abbreviations

ACOD1: Aconitate decarboxylase 1; AGE: Advanced glycation end products; AIM2: Absent in melanoma 2; ASC: Apoptotic speck protein; ATM: Ataxia telangiectasia mutated; ATF4: Activating Transcription Factor 4; CCF: Cytosolic chromatin fragment; cGAS: Cyclic GMP-AMP synthase; cGAMP: 2′3′cyclic GMP–AMP; CTL: Cytotoxic T lymphocyte; CTT: C-terminal tail; DC: Diabetic cardiomyopathy; DFU: diabetic foot ulcers; DN: Diabetic nephropathy; DNA-PK: DNA-dependent protein kinase; DNMT1: DNA Methyltransferase 1; dsDNA: Double-stranded DNA; DWH: Diabetic wound healing; FFA: Free fatty acid; eIF2α: Eukaryotic translation initiation factor 2α; ER: endoplasmic reticulum; ERS: Endoplasmic reticulum stress; GBPs: Guanylate-binding proteins; GSDMD: Gasdermin D; IFI16: Interferon-inducible 16; IFN: Interferon; IL, Interleukin; IKK: Inhibitor of kappa B kinase; IRF3: Interferon regulatory factor 3; IRE1: Inositol- requiring enzyme 1; JAK: Janus kinase; LAP: LC3-associated phagocytosis; LBD: Ligand-binding domain; LB1: Laminin B1; LC3: Microtubule-associated protein 1A/1B-light chain 3; LPS: Lipopolysaccharide; MAFLD: Metabolic dysfunction-associated fatty liver disease; Metrnl: Meteor-like hormone; MGO: Methylglyoxal; mPTP: Mitochondrial permeability transition pore; mt: Mitochondrial; NAFLD: Non-alcoholic fatty liver disease; NF-κB: Nuclear factor kappa-B; NIK: NF-κB-inducing kinase; NLRP3: NOD-like receptor thermal protein domain associated protein 3; Nrf2: Nuclear factor erythroid 2-related factor 2; OXPHOS: Oxidative phosphorylation; PARP-1: Poly (ADP-ribose) polymerase 1; PERK: Protein kinase RNA-like endoplasmic reticulum kinase; ROS: Reactive oxygen species; Rb: Retinoblastoma SASP: Senescence-associated secretory phenotype; SOCS1: Cytokine signal transduction inhibitor 1; STAT: Signal transducer and activator of transcription; STING: Stimulator of interferon genes; STIM1: STING and protein-matrix interacting molecule 1; TBK1: TANK-binding kinase 1; TBM: TBK1-binding motif; TRAF6: TNF receptor associated factor 6; TLR4: Toll-like receptor 4; TYK2: Tyrosine kinase 2; T2D: Type 2 diabetes; ULK1: UNC-52-like kinase 1; UPR: Unfolded-protein response; USP18: Ubiquitin-specific peptidase 18.

## Conflicts of interest

None declared.

## Funding

This work was supported by the National Natural Science Foundation of China (81960741, 82160770), the Guizhou Provincial Natural Science Foundation (QKH-J-2020-1Z070), Outstanding Young Scientific and Technological Talents Project of Guizhou Province (2021–5639), scholarships from the China Scholarship Council (No. CSC-202008520012).

## Authors’ contributions

WH and XM are the main writers of the review, completing the collection and analysis of relevant literature and writing of the first draft of the paper. XW, YeL, JD, YequiL and FH participated in the analysis and collation of literature. XN reviewed the manuscript, making a substantial, direct and intellectual contribution to the work, and approved it for publication (supervisor).
